# Transformer Architecture and Attention Mechanisms in Genome Data Analysis: A Comprehensive Review

**DOI:** 10.3390/biology12071033

**Published:** 2023-07-22

**Authors:** Sanghyuk Roy Choi, Minhyeok Lee

**Affiliations:** School of Electrical and Electronics Engineering, Chung-Ang University, Seoul 06974, Republic of Korea; choiroy@cau.ac.kr

**Keywords:** deep learning, transformer model, attention mechanism, genome data, transcriptome data, genomics, bioinformatics, sequence analysis, natural language processing

## Abstract

**Simple Summary:**

The rapidly advancing field of deep learning, specifically transformer-based architectures and attention mechanisms, has found substantial applicability in bioinformatics and genome data analysis. Given the analogous nature of genome sequences to language texts, these techniques initially successful in natural language processing have been applied to genomic data. This review provides an in-depth analysis of the most recent advancements and applications of these techniques to genome data, critically evaluating their advantages and limitations. By investigating studies from 2019 to 2023, this review identifies potential future research areas, thereby encouraging further advancements in the field.

**Abstract:**

The emergence and rapid development of deep learning, specifically transformer-based architectures and attention mechanisms, have had transformative implications across several domains, including bioinformatics and genome data analysis. The analogous nature of genome sequences to language texts has enabled the application of techniques that have exhibited success in fields ranging from natural language processing to genomic data. This review provides a comprehensive analysis of the most recent advancements in the application of transformer architectures and attention mechanisms to genome and transcriptome data. The focus of this review is on the critical evaluation of these techniques, discussing their advantages and limitations in the context of genome data analysis. With the swift pace of development in deep learning methodologies, it becomes vital to continually assess and reflect on the current standing and future direction of the research. Therefore, this review aims to serve as a timely resource for both seasoned researchers and newcomers, offering a panoramic view of the recent advancements and elucidating the state-of-the-art applications in the field. Furthermore, this review paper serves to highlight potential areas of future investigation by critically evaluating studies from 2019 to 2023, thereby acting as a stepping-stone for further research endeavors.

## 1. Introduction

The revolution of deep learning methodologies has invigorated the field of bioinformatics and genome data analysis, establishing a foundation for ground-breaking advancements and novel insights [1,2,3,4,5,6]. Recently, the development and application of transformer-based architectures and attention mechanisms have demonstrated superior performance and capabilities in handling the inherent complexity of genome data. Deep learning techniques, particularly those utilizing transformer architectures and attention mechanisms, have shown remarkable success in various domains such as natural language processing (NLP) [7] and computer vision [8,9,10]. These accomplishments have motivated their rapid adoption into bioinformatics, given the similar nature of genome sequences to language texts. Genome sequences can be interpreted as the language of biology, and thus, tools proficient in handling language data can potentially decipher the hidden patterns within these sequences.

The attention mechanism, first introduced in sequence-to-sequence models [11], has revolutionized how deep learning models handle and interpret data [12,13,14,15,16,17,18]. This technique was designed to circumvent the limitations of traditional recurrent models by providing a mechanism to attend to different parts of the input sequence when generating the output. In the context of genome data, this implies the ability to consider different genomic regions and their relations dynamically during the interpretation process. The attention mechanism computes a weighted sum of input features, where the weights, also known as attention scores, are dynamically determined based on the input data. This mechanism allows the model to focus more on essential or relevant features and less on irrelevant or less important ones.

Inspired by the success of attention mechanisms, the transformer model was proposed as a complete shift from the sequential processing nature of recurrent neural networks (RNNs) and their variants [19,20,21,22]. The transformer model leverages attention mechanisms to process the input data in parallel, allowing for faster and more efficient computations. The architecture of the transformer model is composed of a stack of identical transformer modules, each with two sub-layers: a multi-head self-attention mechanism and a position-wise fully connected feed-forward network. Using this architecture, transformer models can capture the dependencies between inputs and outputs without regard for their distance in the sequence.

The potential of transformer-based architectures and attention mechanisms in genome data analysis is vast and largely unexplored. They present a promising solution to tackle the massive scale and intricate nature of genomic data. The ability to capture long-range dependencies between genomic positions, consider multiple relevant genomic regions simultaneously, and adaptively focus on salient features makes these methods uniquely suited for genomic applications. This review paper seeks to highlight and investigate the innovative applications of these methods in genome data analysis, critically assess their advantages and limitations, and provide future research directions.

The surge of research in this domain has led to a voluminous influx of studies and publications, each contributing new findings, methods, and perspectives. While this rapid proliferation of research is a testament to the field’s dynamism, it also poses a challenge for researchers to keep pace with the advancements. Hence, the necessity for comprehensive review papers that curate, synthesize, and cohesively present these findings is paramount.

This review paper aims to provide a rigorous and up-to-date synthesis of the proliferating literature in this field. Given the swift pace of development in deep learning methodologies, it is critical to continually assess and reflect on the current standing and future direction of the research. This review will serve as a timely resource for both seasoned researchers and newcomers to the field, offering a panoramic view of the recent advancements and elucidating the state-of-the-art applications of transformer architectures and attention mechanisms in genome data analysis.

This review undertakes a systematic and critical assessment of the most recent studies spanning 2019 to 2023. Thoroughly examining these publications aims to provide novel perspectives, detect existing research gaps, and propose avenues for future investigation. Moreover, this review aims to highlight the far-reaching implications and potential benefits associated with the application of advanced deep learning techniques in the analysis of genome data. By investigating these advances, it seeks to inspire and stimulate further research endeavors and technological breakthroughs in the dynamic field of bioinformatics.

## 2. Deep Learning with Transformers and Attention Mechanism

### 2.1. Conventional Architectures of Deep Learning

In recent years, the field of biomedicine has observed a significant upsurge in the application of machine learning and, more particularly, deep learning methods. These advanced techniques have been instrumental in unearthing insights from complex biomedical datasets, enabling progress in disease diagnosis, drug discovery, and genetic research.

Deep learning, or deep neural networks (DNNs), employs artificial neural networks with multiple layers, a feature that makes it remarkably capable of learning complex patterns from large datasets [23]. One of the simplest forms of a neural network is the multilayer perceptron (MLP), which contains an input layer, one or more hidden layers, and an output layer. MLPs are proficient at handling datasets where inputs and outputs share a linear or non-linear relationship. However, they are less effective when dealing with spatial or temporal data, a limitation overcome by more sophisticated deep learning models such as convolutional neural networks (CNNs) [24] and RNNs [25].

CNNs are exceptionally efficient at processing spatial data, such as images, due to their ability to capture local dependencies in data using convolutional layers. In biomedicine, CNNs have proved instrumental in tasks like medical image analysis and tissue phenotyping.

RNNs, including their advanced variant, long short-term memory (LSTM) networks, are designed to handle sequential data by incorporating a memory-like mechanism, allowing them to learn from previous inputs in the sequence. This property makes them valuable in predicting protein sequences or understanding genetic sequences in bioinformatics.

Generative adversarial networks (GANs), a game-changer in the field, consist of two neural networks, the generator and the discriminator, that compete [26,27,28,29,30,31,32]. This unique architecture enables the generation of new, synthetic data instances that resemble the training data, a feature that holds promise in drug discovery and personalized medicine.

Several other variants of deep learning techniques also exist. For instance, graph attention leverages the attention mechanism to weigh the influence of nodes in a graph, playing a crucial role in molecular biology for structure recognition. Residual networks (ResNets) use shortcut connections to solve the problem of vanishing gradients in deep networks, a feature that can be valuable in medical image analysis. AdaBoost, a boosting algorithm, works by combining multiple weak classifiers to create a strong classifier. Seq2Vec is an approach for sequence data processing where the sequence is converted into a fixed-length vector representation. Finally, variational autoencoders (VAE) are generative models that can learn a latent representation of the input data, offering significant potential in tasks like anomaly detection or dimensionality reduction in complex biomedical data.

### 2.2. Transformers and Attention Mechanism

The transformer model represents a watershed moment in the evolution of deep learning models [33]. Distinct from conventional sequence transduction models, which typically involve recurrent or convolutional layers, the transformer model solely harnesses attention mechanisms, setting a new precedent in tasks such as machine translation and natural language processing (NLP).

The principal component of a transformer model is the attention mechanism, and it comes in two forms: self-attention (also referred to as intra-attention) and multi-head attention. The attention mechanism’s core function is to model interactions between different elements in a sequence, thereby capturing the dependencies among them without regard to their positions in the sequence. In essence, it determines the extent to which to pay attention to various parts of the input when producing a particular output.

Self-attention mechanisms operate by creating a representation of each element in a sequence that captures the impact of all other elements in the sequence. This is achieved by computing a score for each pair of elements, applying a softmax function to obtain weights, and then using these weights to form a weighted sum of the original element representations. Consequently, it allows each element in the sequence to interact with all other elements, providing a more holistic picture of the entire sequence.

The multi-head attention mechanism, on the other hand, is essentially multiple self-attention mechanisms, or heads, operating in parallel. Each head independently computes a different learned linear transformation of the input, and their outputs are concatenated and linearly transformed to result in the final output. This enables the model to capture various types of relationships and dependencies in the data.

In addition to the self-attention mechanism, another critical aspect of the transformer architecture is the incorporation of positional encoding. Given that the model itself is permutation-invariant (i.e., it does not have any inherent notion of the order of the input elements), there is a necessity for some method to incorporate information about the position of the elements within the sequence. Positional encoding serves this purpose.

Positional encodings are added to the input embeddings at the bottoms of the encoder and decoder stacks. These embeddings are learned or fixed, and their purpose is to inject information about the relative or absolute positions of the words in the sequence. The addition of positional encodings enables the model to make use of the order of the sequence, which is critical for understanding structured data like language.

One common approach to positional encoding is to use sine and cosine functions of different frequencies. With this approach, each dimension of the positional encoding corresponds to a sine or cosine function. These functions have a wavelength that forms a geometric progression from 2π to 10,000 × 2π.

One of the key advantages of the transformer model is its ability to handle long-range dependencies in the data, an aspect where traditional RNNs and CNNs may struggle due to their sequential nature. By allowing all elements in the sequence to interact simultaneously, transformers alleviate the need for compressing all information into a fixed-size hidden state, which often leads to information loss in long sequences.

Additionally, transformers also introduce the concept of position encoding to counter the absence of inherent positional information in attention mechanisms. This is crucial, especially in tasks where the order of the elements carries significant information.

The transformer’s self-attention mechanism involves three crucial components: the query (Q), key (K), and value (V). These components originate from the input representations and are created by multiplying the input by the respective learned weight matrices. Each of these components carries a unique significance in the attention mechanism.

In detail, the query corresponds to the element for which we are trying to compute the context-dependent representation. The key relates to the elements that we are comparing the query against to determine the weights. Finally, the value is the element that gets weighted by the attention score (resulting from the comparison of the query with the key) to generate the final output.

The self-attention mechanism operates by calculating an attention score for a pair of query and key. It does so by taking their dot product and then applying a softmax function to ensure that the weights fall into the range of zero and one and sum to one. This provides a normalized measure of importance, or attention, that the model assigns to each element when encoding a particular element.

Following the calculation of attention scores, the model computes a weighted sum of the value vectors, where the weights are given by the attention scores. This operation results in the context-sensitive encoding of each element, where the context depends on all other elements in the sequence. Such encodings are then used as inputs to the next layer in the transformer model.

**Figure 1:**
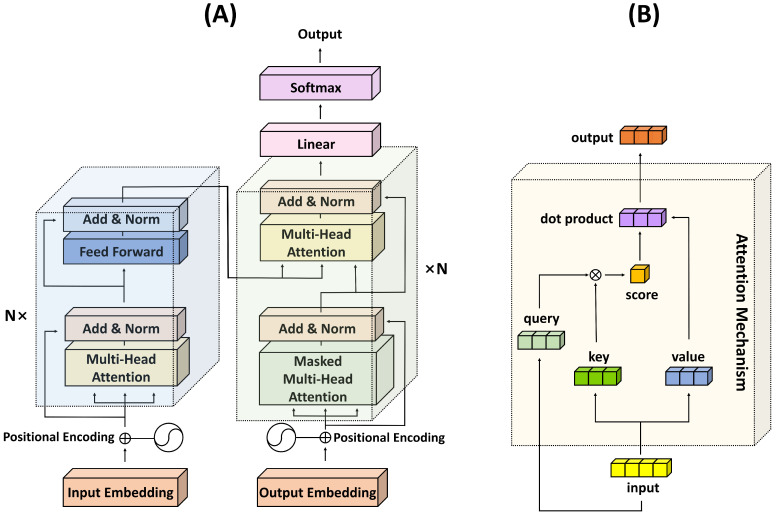
Illustration of the transformer architecture and the attention mechanism. (**A**) Transformer structure; (**B**) Attention mechanism.

The use of the Q, K, and V matrices allows the model to learn to focus on different aspects of the input data and enables it to discern which pieces of information are critical when encoding a particular element. As such, the transformer’s attention mechanism brings a significant degree of flexibility and power to the model, allowing it to handle a wide variety of tasks in an efficient and effective manner. The structures of the transformer architecture and the attention mechanism are depicted in Figure 1.

## 3. Methods

### 3.1. Publication Selection Process

The paper selection process was designed to ensure the inclusion of high-quality research contributions pertinent to our review’s focal area. To achieve this, we primarily leveraged algorithmic approaches relying on the academic search engine, Web of Science (WOS). We carefully chose our search keywords, focusing on core terminologies such as “deep learning transformer”, “attention method”, “RNAs”, and “genome data”. This meticulous selection of search keywords was instrumental in identifying relevant articles for inclusion in our review.

To present a structured overview of the transformer architecture and the attention mechanism in the context of genomic data, we classified the selected papers according to research topic, i.e., the specific application of transformers and attention methods. This classification aims to contribute to a comprehensive understanding of the intersection of deep learning with transformers and attention mechanisms for genomic data. It accentuates the comprehension of various methodologies employed within the field. For a concise summary of the reviewed papers, refer to Table 1. We acknowledge that several papers could fit multiple categories, but for the purpose of this review, each paper was classified under a single category that best captures the paper’s core theme.

Our review focuses solely on peer-reviewed journal articles, knowingly excluding preprints and conference papers, despite their abundance in the field. We enforced this criterion to uphold the reliability and validity of the review, thereby ensuring that only studies subjected to rigorous peer-review scrutiny were included. We aimed to maintain the novelty and originality of the review and, thus, intentionally excluded specific types of articles, such as review articles and perspectives. The objective was to emphasize primary research-based studies as per our review’s intent.

We limited our review’s temporal span to articles published from 2019 to 2023. This constraint ensures that our review remains concurrent and relevant, providing a comprehensive understanding of the most recent advancements and trends in the field of deep learning for genomic data. It is noteworthy that our review focuses solely on peer-reviewed journal articles. This decision was driven by two main factors: First, the peer review process is a crucial mechanism to uphold the quality and reliability of the scientific literature by subjecting research to rigorous examination by domain experts. Second, peer-reviewed journals are traditionally deemed reliable and trusted sources for publishing scientifically sound and influential research.

We carried out data collection for 2023 up until May, aligning with our current schedule, thereby ensuring that the review’s currency aligns with the field’s latest developments. During the data collection process, we compiled information on the number of citations and publication logs for each selected article. These data were paramount for evaluating the scope, impact, and acceptance of the research within the scientific community. We shall analyze these data in the subsequent sections of this review.

Certain studies were excluded based on specific criteria. Review articles were not considered due to our focus on primary research. Studies employing only machine learning methodologies without deep learning elements were also excluded. Furthermore, papers that did not directly relate to genomic data, such as those focusing on image segmentation, were left out, despite the general applicability of the attention mechanism to image data. Hence, such image-related studies were manually removed from our review.

### 3.2. Journals of Published Papers

Table 2 illustrates the distribution of published articles focusing on the application of the transformer architecture and the attention mechanism for genome data, across a variety of scientific journals.

It is evident from Table 2 that ‘Briefings in Bioinformatics’ has the highest number of publications (20), constituting 16.1% of the total studies in this domain. The ‘Bioinformatics’, ‘BMC Bioinformatics’, and ‘Frontiers in Genetics’ journals follow closely, each contributing 7.3% of the total publications. Journals such as ‘PLOS Computational Biology’, ‘Nature Communications’, and ‘Interdisciplinary Sciences-Computational Life Sciences’ account for about 3.2% each.

Furthermore, there is a considerable portion of articles (27.4%) distributed in various other journals, each contributing fewer than two publications. These results exhibit a wide dissemination of research on this topic across various journals, suggesting cross-disciplinary interest and influence of transformer architecture and attention mechanism applications within the field of genome data analysis.

### 3.3. Year-Wise Analysis of Publications

**Figure 2:**
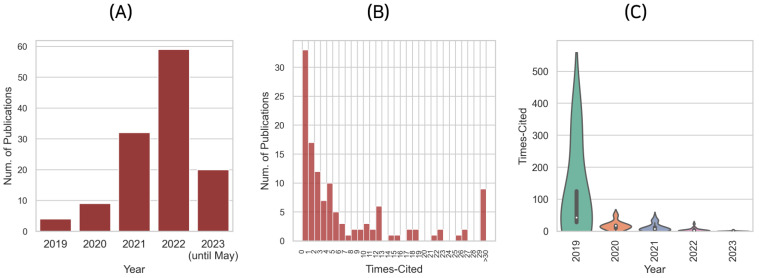
Distribution Patterns of Publication Years and Citation Frequencies (**A**) Distribution of Publication Years. (**B**) Distribution of Citation Frequencies. (**C**) Relationship between Citations and Publication Year.

As illustrated in Figure 2A, the trend of publications on the use of the transformer architecture and the attention mechanism for genome data shows a significant increase over the past few years. The growth and intensity of publications, year after year, illustrate the fast-emerging interest and intensive research activity in this field.

In 2019, the number of publications was relatively small, with only four documented studies, indicating the nascent stage of research in this area. However, the number of publications experienced a substantial rise by more than double, to nine, in 2020. This indicates the field’s emerging development, as more research communities started recognizing the transformative potential of transformer architectures and attention mechanisms for genome data analysis.

The year 2021 marked a significant breakthrough in this field, with 32 publications, more than three times as many as in 2020. This sudden surge can be attributed to the maturation of the methodologies and the growing acknowledgment of their utility and effectiveness in genome data interpretation.

In 2022, the research activity peaked with a record high of 59 publications, indicating a major turning point and signifying the field’s transition into a more mature phase. The proliferation of these techniques in genome data analysis could be attributed to their profound ability to handle large genomic datasets and generate meaningful biological insights.

In 2023, up until May, there have already been 20 publications, indicating a continued strong interest in the field. Despite being only partway through the year, the number of publications has reached approximately one-third of the total for 2022, suggesting that the momentum of research in this area is expected to continue.

The upward trend in the number of publications over the years signifies the growing acknowledgment and adoption of transformer architecture and attention mechanism techniques in genome data analysis. It underscores the importance of further research to leverage these promising deep learning methodologies for more advanced, precise, and insightful interpretation of complex genomic data.

### 3.4. Analysis of Citation Distribution

The citation distribution of the reviewed papers provides insightful data about their scholarly impact and recognition within the academic community. As depicted in Figure 2B, which illustrates the histogram of citations, and Figure 2C, which represents the correlation between the number of citations and the publication year of papers, there is a notable pattern in citation distribution.

The median number of citations is 2, and the mean is 9.7, suggesting a positively skewed distribution of citations. This skewness indicates that while most papers receive few citations, a minority of papers are highly cited, which considerably raises the mean. It is noteworthy that a large number of studies have not been cited yet, primarily because they have been recently published and have not had adequate time for review. This scenario underscores the significance of the present review, which aims to provide a thorough examination of these studies.

Considering the incomplete citation data for 2023, it is apparent that almost every paper published this year has not been cited yet, with a median citation count of zero. This observation aligns with the expected academic trend where newer publications generally have fewer citations due to the time lag inherent in the citation process.

However, earlier publications exhibit a higher citation count, signifying their broader impact and established status in the field. For instance, the median citation count for the papers published in 2019 and 2020 is 42 and 17, respectively. This shows a substantial scholarly impact, demonstrating that the topic reviewed here is of considerable interest and value to the research community.

In this regard, a few highly cited papers have made a particularly significant impact on the field. For example, the work by Armenteros et al. [141], which introduced TargetP 2.0, a state-of-the-art method to identify N-terminal sorting signals in proteins using deep learning, has garnered significant attention, with 333 citations to date. The attention layer of their deep learning model highlighted that the second residue in the protein, following the initial methionine, has a strong influence on classification, a feature not previously emphasized. This highlights how deep learning methods can generate novel insights into biological systems.

Another influential paper is the work by Manica et al. [137], which proposed a novel architecture for interpretable prediction of anticancer compound sensitivity using a multi-modal attention-based convolutional encoder. This work received 56 citations, and its predictive model significantly outperformed the previous state-of-the-art model for drug sensitivity prediction. The authors also provided a comprehensive analysis of the attention weights, further demonstrating the interpretability of the approach.

Lastly, the study by Angenent-Mari et al. [74], which used deep learning to predict the behavior of engineered RNA elements known as toehold switches, also stands out. With 50 citations, this work showed that DNNs trained on nucleotide sequences vastly outperformed previous models based on thermodynamics and kinetics.

These highly cited works underscore the transformative potential of deep learning methods, particularly those leveraging the transformer architecture and attention mechanisms, in enhancing our understanding of biological systems and in advancing predictive modeling in biomedicine. The citation distribution reflects the temporal dynamics of the field’s influence and the increasing recognition of deep learning with transformer architecture and attention mechanism techniques in genome data analysis. Further reviews and analyses of recent papers are required to stimulate discussion and increase their visibility and impact within the academic community.

## 4. Overview of Recent Studies in Transformer Architectures and Attention Mechanisms for Genome Data

### 4.1. Sequence and Site Prediction

In pre-miRNA prediction, Raad et al. [34] introduced miRe2e, a deep learning model based on transformers. The model demonstrated a ten-fold improvement in performance compared to existing algorithms when validated using the human genome. Similarly, Zeng et al. [38] introduced 4mCPred-MTL, a multi-task learning model coupled with a transformer for predicting 4mC sites across multiple species. The model demonstrated a strong feature learning ability, capturing better characteristics of 4mC sites than existing feature descriptors.

Several studies have leveraged deep learning for RNA–protein binding preference prediction. Shen et al. [35] developed a model based on a hierarchical LSTM and attention network which outperformed other methods. Du et al. [42] proposed a deep multi-scale attention network (DeepMSA) based on CNNs to predict the sequence-binding preferences of RNA-binding proteins (RBPs). Pan et al. [43] developed a deep learning model, CRMSNet, that combined CNN, ResNet, and multi-head self-attention blocks to predict RBPs for RNA sequences.

The work by Sun et al. [68] presents a deep learning tool known as PrismNet, designed for predicting RBP interactions, which are integral to RNA function and cellular regulation. This tool stands out as it was built to reflect the dynamic and condition-dependent nature of RBP–RNA interactions, in contrast to existing tools that primarily rely on RNA sequences or predicted RNA structures. The study proposed PrismNet by integrating experimental in vivo RNA structure data with RBP binding data from seven different cell types. This method enables accurate prediction of dynamic RBP binding across diverse cellular conditions.

An important aspect that distinguishes PrismNet is the application of an attention mechanism that identifies specific RBP-binding nucleotides computationally. The study found enrichment of structure-changing variants (termed riboSNitches) among these dynamic RBP-binding sites, potentially offering new insights into genetic diseases associated with dysregulated RBP bindings. Thus, PrismNet provides a method to access previously inaccessible layers of cell-type-specific RBP–RNA interactions, potentially contributing to our understanding and treatment of human diseases. Despite its merits, PrismNet also has potential limitations. For example, the effectiveness of PrismNet relies heavily on the quality and quantity of experimental in vivo RNA structure data and RBP-binding data. This dependence could limit its usefulness in scenarios where such extensive datasets are not available or are incomplete. Furthermore, while PrismNet uses an attention mechanism to identify exact RBP-binding nucleotides, interpreting these attention scores in the biological context may not be straightforward, requiring additional investigation or expertise.

Li et al. [36] proposed an ensemble deep learning model called m6A-BERT-Stacking to detect m6A sites in various tissues of three species. The experimental results demonstrated that m6A-BERT-Stacking outperformed most existing methods based on the same independent datasets. Similarly, Tang et al. [41] presented Deep6mAPred, a deep learning method based on CNN and Bi-LSTM for predicting DNA N6-methyladenosine sites across plant species.

For promoter recognition, Ma et al. [37] proposed a deep learning algorithm, DeeProPre. The model demonstrated high accuracy in identifying the promoter region of eukaryotes. Mai et al. [39] employed and compared the performance of popular NLP models, including XLNET, BERT, and DNABERT, for promoter prediction in freshwater cyanobacterium Synechocystis sp. PCC 6803 and Synechococcus elongatus sp. UTEX 2973.

In predicting RNA solvent accessibility, Huang et al. [45] proposed a sequence-based model using only primary sequence data. The model employed modified attention layers with different receptive fields to conform to the stem-loop structure of RNA chains. Fan et al. [62] proposed a novel computational method called M(2)pred for accurately predicting the solvent accessibility of RNA. The model utilized a multi-shot neural network with a multi-scale context feature extraction strategy.

To predict transcription factor binding sites, Bhukya et al. [56] proposed two models, PCLAtt and TranAtt. The model outperformed other state-of-the-art methods like DeepSEA, DanQ, TBiNet, and DeepATT in the prediction of binding sites between transcription factors and DNA sequences. Cao et al. [51] proposed DeepARC, an attention-based hybrid approach that combines a CNN and an RNN for predicting transcription factor binding sites.

Muneer et al. [57] proposed two deep hybrid neural network models, namely GCN_GRU and GCN_CNN, for predicting RNA degradation from RNA sequences. In the prediction of RNA degradation, He et al. [52] introduced RNAdegformer, a model architecture for predicting RNA degradation. RNAdegformer outperformed previous best methods at predicting degradation properties at nucleotide resolution for COVID-19 mRNA vaccines.

In the identification of pseudouridine (psi) sites, Zhuang et al. [44] developed PseUdeep, a deep learning framework for identifying psi sites in three species: H. sapiens, S. cerevisiae, and M. musculus. The model uses a modified attention mechanism with different receptive fields to conform to the stem-loop structure of RNA chains.

In the prediction of miRNA-disease associations, Zhang et al. [54] developed the Deep Attentive Encoder–Decoder Neural Network (D-AEDNet) to identify the location of transcription factor binding sites (TFBSs) in DNA sequences. Xie et al. [61] presented a new computational method based on positive point-wise mutual information (PPMI) and an attention network to predict miRNA-disease associations (MDAs), called PATMDA. Liang et al. [59] developed a deep learning model, DeepEBV, to predict Epstein–Barr virus (EBV) integration sites. The model leverages an attention-based mechanism to learn local genomic features automatically.

Recent studies have shown a growing interest in utilizing attention mechanisms for analyzing genome data. Attention-based models have gained popularity due to their ability to capture informative patterns and long-range dependencies in genomic sequences. These models have been applied to various tasks, including sequence and site prediction, RNA-protein binding preference prediction, survival prediction, and identification of functional elements in the genome. The use of attention mechanisms in these studies has demonstrated improved performance and accuracy, highlighting the effectiveness of this approach in extracting meaningful information from genome data.

### 4.2. Gene Expression and Phenotype Prediction

Deep learning models have been extensively employed to predict gene expression and phenotypes, demonstrating significant improvements over traditional methods. These models have been particularly effective in capturing complex gene–gene and gene–environment interactions and integrating diverse types of genomic and epigenomic data.

A particularly noteworthy study in gene expression and phenotype prediction is that of Angenent-Mari et al. [74]. Their work explores the application of DNNs for the prediction of the function of toehold switches, which serve as a vital model in synthetic biology. These switches, engineered RNA elements, can detect small molecules, proteins, and nucleic acids. However, the prediction of their behavior has posed a considerable challenge—a situation that Angenent-Mari and colleagues sought to address through enhanced pattern recognition from deep learning.

The methodology employed by the authors involved the synthesis and characterization of a dataset comprising 91,534 toehold switches, spanning 23 viral genomes and 906 human transcription factors. The DNNs trained on these nucleotide sequences notably outperformed prior state-of-the-art thermodynamic and kinetic models in the prediction of the toehold switch function. Further, the authors introduced human-understandable attention-visualizations (VIS4Map) which facilitated the identification of successful and failure modes. The network architecture comprised MLP, CNN, and LSTM networks trained on various inputs, including one-hot encoded sequences and rational features. An ensemble MLP model was also proposed, incorporating both the one-hot encoded sequences and rational features.

The advantages of this method are manifold. The authors leveraged deep learning to predict the function of toehold switches, a task that had previously presented considerable challenges. The outperformance of prior state-of-the-art models is a testament to the efficacy of the proposed approach. Furthermore, the inclusion of VIS4Map attention-visualizations enhances the interpretability of the model, providing valuable insights into the model’s workings and facilitating the identification of areas of success and those that need improvement. Despite these significant strides, the methodology also bears certain limitations. The training process is computationally demanding, necessitating high-capacity hardware and graphic processing units which may not be accessible to all researchers. Furthermore, as with any model, the generalizability of this approach to other classes of RNA or DNA elements remains to be validated. It is also worth noting that while the model outperforms previous models, there is still considerable room for improvement, as the highest R-squared value achieved was 0.70, indicating that the model could explain 70% of the variability in the data.

A key area of focus has been the prediction of gene expression based on histone modifications. Lee et al. [70] developed Chromoformer, a transformer-based deep learning architecture considering large genomic windows and three-dimensional chromatin interactions. Similarly, Chen et al. [71] introduced TransferChrome, a model that uses a densely connected convolutional network and self-attention layers to aggregate global features of histone modification data. Liao et al. [73] also proposed a hybrid convolutional and bi-directional long short-term memory network with an attention mechanism for this task. These models have demonstrated their ability to predict gene expression levels based on histone modification signals accurately.

Several studies have also focused on predicting gene expression and phenotypes based on other genomic and epigenomic data types. For instance, Zhang et al. [69] developed T-GEM, an interpretable deep learning model for gene-expression-based phenotype predictions. Kang et al. [72] proposed a multi-attention-based deep learning model that integrates multiple markers to characterize complex gene regulation mechanisms. These models have shown their ability to integrate diverse data types and capture complex interactions, leading to improved prediction performance.

Several studies have also focused on the prediction of specific types of phenotypes. For instance, Lee et al. [79] proposed BP-GAN, a model that uses generative adversarial networks (GANs) combined with an attention mechanism for predicting RNA Branchpoints (BPs). These studies have shown the potential of deep learning models in predicting specific types of phenotypes.

Recent studies have focused on utilizing deep learning models with attention mechanisms to predict gene expression and phenotypes based on diverse genomic and epigenomic data. These models have shown improvements over traditional methods by capturing complex gene–gene and gene–environment interactions and integrating various data types. Specifically, attention-based models have been employed to predict gene expression levels using histone modification data, such as Chromoformer [70], TransferChrome [71], and a hybrid convolutional and bi-directional LSTM network [73]. Additionally, researchers have explored the prediction of specific phenotypes, such as toehold switch functions [74] and RNA Branchpoints [79], showcasing the versatility and potential of deep learning with attention mechanisms in gene expression and phenotype prediction.

### 4.3. ncRNA and circRNA Studies

The application of deep learning models, particularly those incorporating transformer architectures and attention mechanisms, has been extensively explored in the study of non-coding RNAs (ncRNAs) and circular RNAs (circRNAs). These models have shown promising results in predicting ncRNA-disease associations, lncRNA–protein interactions, and circRNA-RBP interactions, among other tasks.

Yang et al. [93] presented a novel computational method called iCircRBP-DHN that leverages a deep hierarchical network to distinguish circRNA–RBP-binding sites. The core of this approach is a combination of a deep multi-scale residual network and bidirectional gated recurrent units (BiGRUs) equipped with a self-attention mechanism. This architecture simultaneously extracts local and global contextual information from circRNA sequences. The study proposed two novel encoding schemes to enrich the feature representations. The first, KNFP (K-tuple Nucleotide Frequency Pattern), is designed to capture local contextual features at various scales, effectively addressing the information insufficiency issue inherent in conventional one-hot representation. The second, CircRNA2Vec, is based on the Doc2Vec algorithm and aims to capture global contextual features by modeling long-range dependencies in circRNA sequences. This method treats sequences as a language and maps subsequences (words) into distributed vectors, which contribute to capturing the semantics and syntax of these sequences. The effectiveness of iCircRBP-DHN was validated on multiple circRNAs and linear RNAs datasets, and it showed superior performance over state-of-the-art algorithms.

While iCircRBP-DHN exhibits several advantages, it also presents potential limitations. The method’s strengths include its ability to model both local and global contexts within sequences, its robustness against numerical instability, and its scalability, demonstrated by the performance on extensive datasets. However, the method’s performance is heavily reliant on the quality of sequence data and the effectiveness of the CircRNA2Vec and KNFP encoding schemes, which might not capture all nuances of circRNA–RBP interactions. While the self-attention mechanism can provide some insights into what the model deems important, it might not provide a full explanation of the reasoning behind the model’s predictions.

Several studies have focused on predicting lncRNA–disease associations. Liu et al. [83] developed a dual attention network model, which uses two attention layers, for this task, outperforming several latest methods. Similarly, Gao and Shang [89] proposed a new computational model, DeepLDA, which used DNNs and graph attention mechanisms to learn lncRNA and drug embeddings for predicting potential relationships between lncRNAs and drug resistance. Fan et al. [97] proposed GCRFLDA, a novel lncRNA–disease association prediction method based on graph convolutional matrix completion. Sheng et al. [98] developed VADLP, a model designed to predict lncRNA–disease associations using an attention mechanism. These models have demonstrated their ability to accurately predict lncRNA–disease associations, providing valuable insights into the roles of lncRNAs in disease development and progression.

In addition to predicting lncRNA–disease associations, deep learning models have also been used to predict lncRNA–protein interactions. Song et al. [84] presented an ensemble learning framework, RLF-LPI, for predicting lncRNA–protein interactions. Wekesa et al. [85] developed a graph representation learning method, GPLPI, for predicting plant lncRNA–protein interactions (LPIs) from sequence and structural information. These models have shown their ability to capture dependencies between sequences and structures, leading to improved prediction performance.

In the task of distinguishing circular RNA (circRNA) from other long non-coding RNA (lncRNA), Liu et al. [101] proposed an attention-based multi-instance learning (MIL) network. The model outperformed state-of-the-art models in this task.

Several studies have also focused on the prediction of circRNA–RBP interactions. Wu et al. [86] proposed an RBP-specific method, iDeepC, for predicting RBP-binding sites on circRNAs from sequences. Yuan and Yang [90] developed a deep learning method, DeCban, to identify circRNA–RBP interactions. Niu et al. [99] proposed CRBPDL, a calculation model that employs an Adaboost integrated deep hierarchical network to identify binding sites of circular RNA–RBP. These models have demonstrated their ability to accurately predict circRNA–RBP interactions, providing valuable insights into the roles of circRNAs in post-transcriptional regulation. Guo et al. [102] proposed a deep learning model, circ2CBA, for predicting circRNA–RBP-binding sites. The model achieved an AUC value of 0.8987, outperforming other methods in predicting the binding sites between circRNAs and RBPs.

In addition to predicting interactions, deep learning models have also been used to predict and interpret post-transcriptional RNA modifications and ncRNA families. Song et al. [91] presented MultiRM, a method for the integrated prediction and interpretation of post-transcriptional RNA modifications from RNA sequences. Chen et al. [92] developed ncDENSE, a deep-learning-model-based method for predicting and interpreting non-coding RNAs families from RNA sequences. These models have shown their ability to accurately predict and interpret RNA modifications and ncRNA families, providing valuable insights into the roles of these modifications and families in gene regulation.

Several studies have also focused on predicting circRNA–disease associations. Li et al. [96] proposed a method called GATGCN that utilizes a graph attention network and a convolutional graph network (GCN) to explore human circRNA–disease associations based on multi-source data. Wang et al. [95] proposed CDA-SKAG, a deep learning model for predicting circRNA–disease associations. Li et al. [94] introduced a deep learning model, GGAECDA, to predict circRNA–disease associations. These models have demonstrated their ability to accurately predict circRNA–disease associations, providing valuable insights into the roles of circRNAs in disease development and progression.

Recent studies have focused on utilizing deep learning models with transformer architectures and attention mechanisms for the analysis of ncRNAs and circRNAs. These models have shown promise in various tasks, including the prediction of ncRNA–disease associations, lncRNA–protein interactions, circRNA–RBP interactions, and the identification of RNA modifications and ncRNA families. The integration of attention mechanisms in these models has improved prediction accuracy and facilitated the interpretation of complex interactions and patterns in genomic data.

### 4.4. Transcription Process Insights

In recent advancements, deep learning, specifically attention mechanisms and transformer models, have been significantly employed in decoding the transcription process of genome data. Clauwaert et al. [103], Park et al. [108], and Han et al. [105] have proposed transformative models centered on transcription factor (TF)-binding site prediction and characterization.

As one of the specific examples, Yan et al. [109] introduced an innovative deep learning framework for circRNA–RBP-binding site discrimination, referred to as iCircRBP-DHN, Integrative Circular RNA–RBP-binding sites Discrimination by Hierarchical Networks. They addressed common issues with previous computational models, such as poor scalability and numerical instability, and developed a transformative method that amalgamates local and global contextual information via deep multi-scale residual network BiGRUs with a self-attention mechanism.

One of the key advantages of this approach is the fusion of two encoding schemes, CircRNA2Vec and the K-tuple nucleotide frequency pattern, which allows for the representation of different degrees of nucleotide dependencies, enhancing the discriminative power of feature representations. The robustness and superior performance of this method were evidenced through extensive testing on 37 circRNA datasets and 31 linear RNA datasets, where it outperformed other state-of-the-art algorithms.

Clauwaert et al. [103] used a transformer-based neural network framework for prokaryotic genome annotation, primarily focusing on Escherichia coli. The study emphasized that a substantial part of the model’s subunits or attention heads were attuned to identify transcription factors and characterize their binding sites and consensus sequences. This method opened the door to understanding well-known and possibly novel elements involved in transcription initiation. Furthering the area of TF-binding site prediction, Park et al. [108] introduced TBiNet, an attention-based deep neural network model that quantitatively outperformed state-of-the-art methods and demonstrated increased efficiency in discovering known TF-binding motifs. This study aimed to augment the interpretability of TF-binding site prediction models, an aspect critical to comprehending gene regulatory mechanisms and identifying disease-associated variations in non-coding regions. Han et al. [105] proposed MAResNet, a deep learning method combining bottom-up and top-down attention mechanisms and a ResNet to predict TF-binding sites. The model’s robust performance on a vast test dataset reaffirmed the potency of attention mechanisms in capturing complex patterns in genomic sequences.

Another interesting application of deep learning is seen in the study by Feng et al. [104], where they developed a model, PEPMAN, that predicts RNA polymerase II pausing sites based on NET-seq data, which are data from a high-throughput technique used to precisely map and quantify nascent transcriptional activity across the genome. PEPMAN utilized attention mechanisms to decipher critical sequence features underlying the pausing of Pol II. Their model’s predictions, in association with various epigenetic features, delivered enlightening insights into the transcription elongation process.

Regarding RNA localization, Asim et al. [107] developed EL-RMLocNet, an explainable LSTM network for RNA-associated multi-compartment localization prediction, utilizing a novel GeneticSeq2Vec statistical representation learning scheme and an attention mechanism. This model surpassed the existing state-of-the-art predictor for subcellular localization prediction.

In predicting RBP-binding sites, Song et al. [110] proposed AC-Caps, an attention-based capsule network. The model achieved high performance, with an average AUC of 0.967 and an average accuracy of 92.5%, surpassing existing deep-learning models and proving effective in processing large-scale RBP-binding site data.

Tao et al. [106] presented a novel application in oncology; they developed an interpretable deep learning model, CITRUS, which inferred transcriptional programs driven by somatic alterations across different cancers. CITRUS utilized a self-attention mechanism to model the contextual impact of somatic alterations on TFs and downstream transcriptional programs. It revealed relationships between somatic alterations and TFs, promoting personalized therapeutic decisions in precision oncology.

Deep learning models with attention mechanisms and transformer architectures have emerged as powerful tools for gaining insights into the transcription process and decoding genome data. These models have been applied to various tasks, such as TF-binding site prediction and characterization. Many studies have proposed transformative models that utilize attention mechanisms to identify TFs, characterize their binding sites, and understand gene regulatory mechanisms. Additionally, deep learning models have been employed to predict RNA polymerase II pausing sites, RNA localization, RBP-binding sites, and transcriptional programs driven by somatic alterations in cancer. These studies highlight the effectiveness of attention mechanisms in capturing complex patterns in genomic sequences and providing valuable insights into the transcription process and gene regulation.

### 4.5. Multi-Omics/Modal Tasks

Exploring and integrating multi-omics and multi-modal data are substantial tasks in understanding complex biological systems. Deep learning methods, particularly attention mechanisms and transformer models, have seen profound advancements and deployments in this regard. Studies by Gong et al. [111], Kayikci and Khoshgoftaar [112], Ye et al. [113], and Wang et al. [115] have extensively utilized such methods for biomedical data classification and disease prediction.

In the study by Kang et al. [114], a comprehensive ensemble deep learning model for plant miRNA–lncRNA interaction prediction is proposed, namely PmliPEMG. This method introduces a fusion of complex features, multi-scale convolutional long short-term memory (ConvLSTM) networks, and attention mechanisms. Complex features, built using non-linear transformations of sequence and structure features, enhance the sample information at the feature level. By forming a matrix from the complex feature vector, the ConvLSTM models are used as the base model, which is beneficial due to their ability to extract and memorize features over time. Notably, the models are trained on three matrices with different scales, thus enhancing sample information at the scale level.

An attention mechanism layer is incorporated into each base model, assigning different weights to the output of the LSTM layer. This attentional layer allows the model to focus on crucial information during training. Finally, an ensemble method based on a greedy fuzzy decision strategy is implemented to integrate the three base models, improving efficiency and generalization ability. This approach exhibits considerable advantages. Firstly, the use of multi-level information enhancement ensures a more comprehensive understanding of the underlying data, increasing the robustness of the method. The greedy fuzzy decision enhances the model’s efficiency and overall generalization ability. Furthermore, the application of attention mechanisms allows the model to focus on the most informative features, improving predictive accuracy.

Gong et al. [111] proposed MOADLN, a multi-omics attention deep learning network, which is adept at exploring correlations within and across different omics datasets for biomedical data classification. This methodology showcased its effectiveness in deep-learning-based classification tasks. Kayikci and Khoshgoftaar [112] proposed AttentionDDI, a gated attentive multi-modal deep learning model for predicting breast cancer by integrating clinical, copy number alteration, and gene expression data. It demonstrated the potential for significant improvements in breast cancer detection and diagnosis, suggesting better patient outcomes. Ye et al. [113] implemented a novel gene prediction method using a Siamese neural network, a deep learning architecture that employs twin branches with shared weights to compare and distinguish similarity or dissimilarity between input samples, containing a lightweight attention module for identifying ovarian cancer causal genes. This approach outperformed others in accuracy and effectiveness. Similarly, Wang et al. [115] proposed a deep neural network model that integrates multi-omics data to predict cellular responses to known anti-cancer drugs. It employs a novel graph embedding layer and attention layer that efficiently combines different omics features, accounting for their interactions.

Chan et al. [116] proposed a deep neural network architecture combining structural and functional connectome data, which refers to the comprehensive mapping and analysis of neural connections within the brain, with multi-omics data for disease classification. They utilized graph convolution layers for the simultaneous modeling of functional Magnetic Resonance Imaging (fMRI) and Diffusion Tensor Imaging (DTI) data, which are neuroimaging techniques used to, respectively, measure blood flow changes and diffusion patterns within the brain; and separate graph convolution layers for modeling multi-omics datasets. An attention mechanism was used to fuse these outputs, highlighting which omics data contributed the most to the classification decision. This approach demonstrated a high efficacy in Parkinson’s disease classification using various combinations of multi-modal imaging data and multi-omics data.

These studies highlight the potential of attention mechanisms and transformer models in decoding complex biological systems and addressing multi-omics and multi-modal challenges in genomics research.

### 4.6. CRISPR Efficacy and Outcome Prediction

The efficacy and outcome prediction of CRISPR-Cas9 gene editing have significantly improved due to the development of sophisticated deep learning models. Several studies, including Liu et al. [118], Wan and Jiang [119], Xiao et al. [120], Mathis et al. [121], Zhang et al. [122], and Zhang et al. [123], have extensively used such models to predict CRISPR-Cas9 editing outcomes, single guide RNAs (sgRNAs) knockout efficacy, and off-target activities, enhancing the precision of gene editing technologies.

The research by Zhang et al. [123] introduced a novel method for predicting on-target and off-target activities of CRISPR/Cas9 sgRNAs. They proposed two deep learning models, CRISPR-ONT and CRISPR-OFFT, which incorporate an attention-based CNN to focus on sequence elements most decisive in sgRNA efficacy. These models offer several key advantages. First, they utilize an embedding layer that applies k-mer encoding to transform sgRNA sequences into numerical values, allowing the CNN to extract feature maps. This technique has been demonstrated to outperform other methods in sequential analysis. Second, these models use attention mechanisms to improve both prediction power and interpretability, focusing on the elements of the input sequence that are the most relevant to the output. This mirrors how RNA-guide Cas9 nucleases scan the genome, enhancing the realism of the model.

Liu et al. [118] presented Apindel, a deep learning model utilizing the GloVe model, a widely used unsupervised learning algorithm that captures the semantic relationships between words by analyzing the global statistical co-occurrence patterns of words within a large corpus. By integrating the GloVe, positional encoding, and a deep learning model embedding BiLSTM and attention mechanism, the proposed model predicts CRISPR-Cas9 editing outcomes by capturing the semantic relationships. It outperformed most advanced models in DNA mutation prediction and provided more detailed prediction categories. In the same vein, Wan and Jiang [119] introduced TransCrispr, a model combining transformer and CNN architectures for predicting sgRNA knockout efficacy in the CRISPR-Cas9 system. The model exhibited superior prediction accuracy and generalization ability when tested on seven public datasets.

Moreover, Xiao et al. [120] proposed AttCRISPR, an interpretable spacetime model for predicting the on-target activity of sgRNA in the CRISPR-Cas system. The model incorporated encoding-based and embedding-based methods using an ensemble learning strategy and achieved a superior performance compared to state-of-the-art methods. Notably, the model incorporated two attention modules, one spatial and one temporal, to enhance interpretability. Similarly, Liu et al. [117] developed an interpretable machine learning model for predicting the efficiency and specificity of the CRISPR-Cas system.

Mathis et al. [121] utilized attention-based bidirectional RNNs to develop PRIDICT, an efficient model for predicting prime editing outcomes. The model demonstrated reliable predictions for small-sized genetic alterations and highlighted the robustness of PRIDICT in improving prime editing efficiencies across various cell types.

In line with off-target activities prediction, Zhang et al. [122] presented a novel model, CRISPR-IP, for effectively harnessing sequence pair information to predict off-target activities within the CRISPR-Cas9 gene editing system. Their methodology integrated CNN, BiLSTM, and the attention layer, demonstrating superior performance compared to existing models.

Recent studies have made significant advancements in predicting the efficacy and outcomes of CRISPR-Cas9 gene editing using deep learning models. These models have demonstrated superior accuracy and performance in predicting CRISPR-Cas9 editing outcomes, sgRNA knockout efficacy, and off-target activities. The integration of attention mechanisms in these models has improved interpretability and provided valuable insights into the mechanisms of CRISPR-Cas9 gene editing.

### 4.7. Gene Regulatory Network Inference

The emergence of deep learning has revolutionized the inference of gene regulatory networks (GRNs) from single-cell RNA-sequencing (scRNA-seq) data, underscoring the utility of transformative machine learning architectures such as the attention mechanism and transformers. Prominent studies, including Lin and Ou-Yang [124], Xu et al. [125], Feng et al. [126], Ullah and Ben-Hur [127], and Xie et al. [128], have utilized these architectures to devise models for GRN inference, highlighting their superior performance compared to conventional methodologies.

The study by Ullah and Ben-Hur [127] presented a novel model, SATORI, for the inference of GRNs. SATORI is a Self-ATtentiOn-based model engineered to detect regulatory element interactions. SATORI leverages the power of deep learning through an amalgamation of convolutional layers and a self-attention mechanism. The convolutional layers, assisted by activation and max-pooling, process the input genomic sequences represented through one-hot encoding. The model further incorporates an optional RNN layer with long short-term memory units for temporal information capture across the sequence.

The multi-head self-attention layer in SATORI is its most pivotal component, designed to model dependencies within the input sequence irrespective of their relative distances. This feature enables the model to effectively capture transcription factor cooperativity. The model is trained and evaluated through a random search algorithm for hyperparameter tuning and the area under the ROC curve for performance measurement. One of the most distinctive features of SATORI is its ability to identify interactions between sequence motifs, contributing to its interpretability. It uses integrated gradients to calculate attribution scores for motifs in a sequence. Changes in these scores after motif mutation can suggest potential interactions. In benchmarking experiments, SATORI demonstrated superior detection rates of experimentally validated transcription factor interactions compared to existing methods without necessitating computationally expensive post-processing.

Lin and Ou-Yang [124] proposed DeepMCL, a model leveraging multi-view contrastive learning to infer GRNs from multiple data sources or time points. DeepMCL represented each gene pair as a set of histogram images and introduced a deep Siamese convolutional neural network with contrastive loss, a loss function commonly used in unsupervised or self-supervised learning tasks that encourages similar samples to be closer in the embedding space while pushing dissimilar samples farther apart; this allows the low-dimensional embedding for each gene pair to be obtained. Moreover, an attention mechanism was employed to integrate the embeddings extracted from different data sources and neighbor gene pairs.

Similarly, Xu et al. [125] presented STGRNS, an interpretable transformer-based method for inferring GRNs from scRNA-seq data. The method leveraged the gene expression motif technique to convert gene pairs into contiguous sub-vectors, which then served as the input for the transformer encoder. Furthermore, Feng et al. [126] introduced scGAEGAT, a multi-modal model integrating graph autoencoders and graph attention networks for single-cell RNA-seq analysis, exhibiting a promising performance in gene imputation and cell clustering prediction.

Xie et al. [128] proposed MVIFMDA, a multi-view information fusion method for predicting miRNA–disease associations. The model employed networks constructed from known miRNA–disease associations and miRNA and disease similarities, processed with a graph convolutional network, followed by an attention strategy to fuse topology representation and attribute representations.

The successful application of deep learning— particularly, attention mechanisms and transformer models—in GRN inference highlights its potential to enhance the precision of gene regulatory network predictions and other genetic analyses. These models have demonstrated superior performance and interpretability, outperforming conventional methods and providing valuable insights into gene regulation and disease mechanisms.

### 4.8. Disease Prognosis Estimation

Deep learning models with transformer architectures and attention mechanisms have seen significant utilization in estimating disease prognosis, demonstrating their efficacy in extracting meaningful patterns from complex genomic data. Among the trailblazing studies in this area include those conducted by Lee [129], Choi and Lee [130], Dutta et al. [131], Xing et al. [132], and Meng et al. [133].

Lee [129] introduced the Gene Attention Ensemble NETwork (GAENET), a model designed for prognosis estimation of low-grade glioma (LGG). GAENET incorporated a gene attention mechanism tailored for gene expression data, outperforming traditional methods and identifying HILS1 as the most significant prognostic gene for LGG. Similarly, Choi and Lee [130] proposed Multi-PEN, a deep learning model that utilizes multi-omics and multi-modal schemes for LGG prognosis. The model incorporated gene attention layers for each data type, such as mRNA and miRNA, to identify prognostic genes, showing robust performance compared to existing models.

The power of self-attention was highlighted by Dutta et al. [131] through their deep multi-modal model, DeePROG, designed to forecast the prognosis of disease-affected genes from heterogeneous omics data. DeePROG outperformed baseline models in extracting valuable features from each modality and leveraging the prognosis of the biomedical data. On the other hand, Xing et al. [132] developed MLA-GNN, a multi-level attention graph neural network for disease diagnosis and prognosis. Their model formatted omics data into co-expression graphs and constructed multi-level graph features, achieving exceptional performance on transcriptomic data from The Cancer Genome Atlas datasets (TCGA-LGG/TCGA-GBM) and proteomic data from COVID-19/non-COVID-19 patient sera.

In a distinct but related context, Meng et al. [133] introduced a novel framework called SAVAE-Cox for survival analysis of high-dimensional transcriptome data. The model incorporated a novel attention mechanism and fully leveraged an adversarial transfer learning strategy, outperforming state-of-the-art survival analysis models on the concordance index. Feng et al. [134] applied a deep learning model with an attention mechanism. The classifier could accurately predict survivals, with area under the receiver operating characteristic (ROC) curves and time-dependent ROCs reaching 0.968 and 0.974 in the training set, respectively.

Taken together, these studies collectively highlight the potential of attention mechanisms in improving disease prognosis estimation, heralding a new paradigm in analyzing genomic data for prognostic purposes. Their efficacy across a range of disease types and data modalities signifies a promising avenue for future research in precision medicine.

### 4.9. Gene Expression-Based Classification

The implementation of deep learning models with transformer architectures and attention mechanisms has significantly improved the classification accuracy based on gene expressions, as presented in numerous studies by Gokhale et al. [135], Beykikhoshk et al. [136], Manica et al. [137], and Lee et al. [138].

Gokhale et al. [135] put forth GeneViT, a vision transformer method, which is a deep learning architecture that applies the principles of self-attention and transformer models to visual data for classifying cancerous gene expressions. This innovative approach started with a dimensionality reduction step using a stacked autoencoder, followed by an improved DeepInsight algorithm, which is a method to transform non-image data to be used for convolution neural network architectures, achieving a remarkable performance edge over existing methodologies, as observed from evaluations on ten benchmark datasets.

Similarly, in the quest to improve breast cancer subtype classification, Beykikhoshk et al. [136] introduced DeepTRIAGE. This deep learning architecture adopted an attention mechanism to derive personalized biomarker scores, thereby allocating each patient with interpretable and individualized biomarker scores. Remarkably, DeepTRIAGE uncovered a significant association between the heterogeneity within luminal A biomarker scores and tumor stage.

In a different application, Manica et al. [137] crafted a novel architecture for the interpretable prediction of anti-cancer compound sensitivity. This model utilized a multi-modal attention-based convolutional encoder and managed to outstrip both a baseline model trained on Morgan fingerprints, a type of molecular fingerprinting technique used in chemoinformatics to encode structural information of molecules, and a selection of encoders based on the Simplified Molecular Input Line Entry System (SMILES), along with previously reported state-of-the-art methodologies for multi-modal drug sensitivity prediction.

Lee et al. [138] developed an innovative pathway-based deep learning model with an attention mechanism and network propagation for cancer subtype classification. The model incorporated graph convolutional networks to represent each pathway and a multi-attention-based ensemble model was used to amalgamate hundreds of pathways. The model demonstrated high classification accuracy in experiments with five TCGA cancer datasets and revealed subtype-specific pathways and biological functions, providing profound insights into the biological mechanisms underlying different cancer subtypes.

These studies highlight the effectiveness and innovative applications of attention mechanisms in genomic data analysis, offering new insights in precision medicine and oncology.

### 4.10. Proteomics

The utilization of deep learning, particularly the incorporation of transformer architectures and attention mechanisms in proteomics, has led to groundbreaking developments in the prediction of protein functionality, as depicted in the studies by Hou et al. [139], Gong et al. [140], Armenteros et al. [141], and Littmann et al. [142].

Hou et al. [139] constructed iDeepSubMito, a deep neural network model designed for the prediction of protein submitochondrial localization. This model employed an inventive graph embedding layer that assimilated interactome data as prior information for prediction. Additionally, an attention layer was incorporated for the integration of various omics features while considering their interactions. The effectiveness of this model was validated by its outperformance of other computational methods during cross-validation on two datasets containing proteins from four mitochondrial compartments.

Meanwhile, Gong et al. [140] proposed an algorithm, iDRO, aimed at optimizing mRNA sequences based on given amino acid sequences of target proteins. Their algorithm involved a two-step process consisting of open reading frame (ORF) optimization and untranslated region (UTR) generation. The former step used BiLSTM-CRF for determining the codon for each amino acid, while the latter step involved RNA-Bart for outputting the corresponding UTR. The optimized sequences of exogenous genes adopted the pattern of human endogenous gene sequences, and the mRNA sequences optimized by their method exhibited higher protein expression compared to traditional methods.

Armenteros et al. [141] showcased TargetP 2.0, a state-of-the-art machine learning model that identifies N-terminal sorting signals in peptides using deep learning. Their model emphasized the second residue’s significant role in protein classification, revealing unique distribution patterns among different groups of proteins and targeting peptides.

Littmann et al. [142] introduced bindEmbed21, a method predicting protein residues binding to metal ions, nucleic acids, or small molecules. This model leveraged embeddings from the transformer-based protein Language Model ProtT5, outperforming MSA-based predictions using single sequences. Homology-based inference further improved performance, and the method found binding residues in over 42% of all human proteins not previously implied in binding. These studies demonstrate the significant potential of transformer architectures and attention mechanisms in deep learning models for precise protein functionality prediction.

### 4.11. Cell-Type Identification

In recent studies, the application of transformer architectures and attention mechanisms in deep learning has brought significant progress to cell-type identification, demonstrating superior performance across various cell types, species, and sequencing depths. The application of transformer architectures and attention mechanisms in deep learning for cell-type identification has seen significant advancements, as evidenced in the studies by Song et al. [143], Feng et al. [144], Buterez et al. [145], and Zhang et al. [146].

Song et al. [143] developed TransCluster, a hybrid network structure that leverages linear discriminant analysis and a modified transformer for enhancing feature learning in single-cell transcriptomic maps. This method outperformed known techniques on various cell datasets from different human tissues, demonstrating high accuracy and robustness.

Feng et al. [144] proposed a directed graph neural network model named scDGAE for single-cell RNA-seq data analysis. By employing graph autoencoders and graph attention networks, scDGAE retained the connection properties of the directed graph and broadened the receptive field of the convolution operation. This model excelled in gene imputation and cell clustering prediction on four scRNA-seq datasets with gold-standard cell labels.

Furthermore, Buterez et al. [145] introduced CellVGAE, a workflow for unsupervised scRNA-seq analysis utilizing graph attention networks. This variational graph autoencoder architecture operated directly on cell connectivity for dimensionality reduction and clustering. Outperforming both neural and non-neural techniques, CellVGAE provided interpretability by analyzing graph attention coefficients, capturing pseudotime and NF-kappa B activation dynamics.

Zhang et al. [146] showcased RefHiC, an attention-based deep learning framework for annotating topological structures from Hi-C, which is a genomic technique that measures the three-dimensional spatial organization of chromatin within the nucleus. Utilizing a reference panel of Hi-C datasets, RefHiC demonstrated superior performance across different cell types, species, and sequencing depths.

### 4.12. Predicting Drug–Drug Interactions

Recent studies have showcased the remarkable progress in predicting drug–drug interactions (DDIs) through the use of deep learning models incorporating transformer architecturesmand attention mechanisms, surpassing classical and other deep learning methods while highlighting significant drug substructures. Deep learning with transformer architectures and attention mechanisms has significantly advanced the prediction of DDIs. Schwarz et al. [147] introduced AttentionDDI, a Siamese self-attention multi-modal neural network that integrates various drug similarity measures derived from drug characteristics. It demonstrated competitive performance compared to state-of-the-art DDI models on multiple benchmark datasets. Similarly, Kim et al. [148] developed DeSIDE-DDI, a framework that incorporates drug-induced gene expression signatures for DDI prediction. This model excelled with an AUC of 0.889 and an Area Under the Precision–Recall (AUPR) of 0.915, surpassing other leading methods in unseen interaction prediction.

Furthermore, Liu and Xie [149] proposed TranSynergy, a knowledge-enabled and self-attention transformer-boosted model for predicting synergistic drug combinations. TranSynergy outperformed existing methods and revealed new pathways associated with these combinations, providing fresh insights for precision medicine and anti-cancer therapies. Wang et al. [150] also developed a deep learning model, DeepDDS, for identifying effective drug combinations for specific cancer cells. It surpassed classical machine learning methods and other deep-learning-based methods, highlighting significant chemical substructures of drugs. Together, these studies highlight the utility of transformer architectures and attention mechanisms in predicting drug–drug interactions, paving the way for further advancements in the field.

### 4.13. Other Topics

Transformer architectures and attention mechanisms have found applications in various genomic research topics, highlighting the versatility of transformer architectures and attention mechanisms in genomics research. For instance, Yu et al. [151] developed IDMIL-III, an imbalanced deep multi-instance learning approach, which excellently predicts genome-wide isoform-isoform interactions, and Yamaguchi and Saito [152] enhanced transformer-based variant effect prediction by proposing domain architecture (DA)-aware evolutionary fine-tuning protocols, which are computational methods that leverage evolutionary algorithms and consider the structural characteristics of protein domains to optimize and refine protein sequence alignments.

On the other hand, Zhou et al. [153] combined convolutional neural networks with transformers in a deep learning model, INTERACT, to predict the effects of genetic variations on DNA methylation levels. Cao et al. [154] presented DeepASmRNA, an attention-based convolutional neural network model, showing promising results for predicting alternative splicing events.

Gupta and Shankar [155] innovatively proposed miWords, a system that treats the genome as sentences composed of words, to identify pre-miRNA regions across plant genomes, achieving an impressive accuracy of 98%. Concurrently, Zhang et al. [156] developed iLoc-miRNA, a deep learning model employing BiLSTM with multi-head self-attention for predicting the location of miRNAs in cells, showing high selectivity for extracellular miRNAs.

Choi and Chae [157] introduced moBRCA-net, a breast cancer subtype classification framework, which significantly improved performance by integrating multiple omics datasets. These studies showcase the versatility and potential of transformer architectures and attention mechanisms in diverse genomic research contexts.

## 5. Discussion

In consideration of the existing literature, it is evident that deep learning models employing transformer architectures and attention mechanisms have shown promising results in analyzing genome data. However, challenges persist, and opportunities for future work are manifold.

### 5.1. Challenges

One of the principal challenges inherent in applying deep learning models to genomic data pertains to the complex structure of these data. Specifically, gene expression data are typically represented as high-dimensional vectors due to the number of genes captured in each sample during the high-throughput sequencing. This representation poses a challenge for conventional data analysis and interpretation methods. Although some studies, such as those by Lee et al. [70] and Chen et al. [71], have made strides in this aspect by proposing novel model architectures or preprocessing techniques, the high-dimensional nature of genomic data remains a challenge.

Another significant challenge is the limited availability of labeled data. In many tasks such as predicting lncRNA–disease associations or circRNA–RBP interactions, the amount of experimentally confirmed positive and negative associations is often insufficient for training deep learning models [83,86]. This can lead to models that are biased towards the majority class and, therefore, provide poor performance on the minority class.

The inherent complexity of biological systems also poses significant challenges. For instance, gene–gene and gene–environment interactions are complex and often non-linear, making them challenging to capture with standard deep learning models [72,74]. Furthermore, genomic and epigenomic data are often heterogeneous, consisting of diverse data types such as sequence data, gene expression data, and histone modification data. Integrating these diverse data types in a unified model can be challenging.

### 5.2. Future Work

One promising direction for future work is to develop novel model architectures that can effectively handle the high-dimensional nature of genomic data. This could involve designing models that can automatically extract relevant features from the data or leveraging techniques such as dimensionality reduction or feature selection. Moreover, the incorporation of biological prior knowledge into the design of these models could help guide the feature extraction process and lead to more interpretable models.

There is also a need for methods that can effectively deal with the limited availability of labeled data in genomics. One promising approach is to leverage unsupervised or semi-supervised learning techniques, which can make use of unlabeled data to improve model performance [158,159,160]. Transfer learning, where a model trained on a large dataset is fine-tuned on a smaller, task-specific dataset, could also be a promising approach for dealing with the scarcity of labeled data [161,162,163].

Addressing the complexity of biological systems could involve developing models that can capture the intricate interactions and non-linear relationships that are typical in biological systems. These models would need to be able to accommodate the heterogeneity of genomic and epigenomic data. Recent work by Kang et al. [72] and Liao et al. [73] points to the potential of multi-modal deep learning models in this regard. Further research is needed to develop and refine such models for various tasks in genomics.

Also, the incorporation of domain knowledge into the models could be another promising approach. By incorporating known biological mechanisms or relationships into the models, we could guide the learning process and make the learned representations more interpretable.

Finally, the emergence of transformer-based models, such as the GPT families, provides an exciting opportunity for future work. These models have shown great promise in natural language processing, and their ability to model long-range dependencies, where distant genomic elements often interact with each other, could be highly beneficial in genomics. Therefore, adapting and applying these transformer-based models to genomic data is a promising direction for future work.

## 6. Conclusions

In the rapidly advancing landscape of bioinformatics, the need for a comprehensive synthesis of the most recent developments and methodologies is essential. This review aims to provide an extensive examination of the transformative use of deep learning, specifically transformer architectures and attention mechanisms, in the analysis of protein–protein interactions. The swift evolution of these computational strategies has significantly enhanced our capacity to process and decipher complex genomic data, marking a new epoch in the field.

The analysis presented herein, drawn from the most recent studies from 2019 to 2023, emphasizes the astounding versatility and superior performance of these deep learning techniques in a multitude of applications. From sequence and site prediction and gene expression and phenotype prediction, to the more complex multi-omics tasks and disease prognosis estimation, deep learning techniques have proven their potential in elucidating hidden patterns and relationships within genomic sequences. Furthermore, the application of transformer architectures and attention mechanisms has not only expedited computations but also improved accuracy and interpretability, ultimately driving the field forward.

Despite the remarkable advancements and successes recorded, it is important to note that the integration of deep learning in genome data analysis is still in its infancy. There remain several challenges and limitations to be addressed, particularly in improving the interpretability of these models and adapting them for use with smaller datasets, often encountered in the domain of genomics. Moreover, with the ever-growing complexity and scale of genomic data, there is a constant demand for even more advanced and efficient computational tools.

Through this review, we hope to provide a platform for researchers to engage with the latest advancements, familiarize themselves with the state-of-the-art applications, and identify potential gaps and opportunities for future exploration. This synthesis, encompassing a wide array of research topics and applications, demonstrates the immense potential and broad applicability of deep learning techniques in bioinformatics.

The integration of deep learning methodologies, particularly transformer architectures and attention mechanisms, into the bioinformatics toolkit has greatly facilitated our understanding of the ’language of biology’. These powerful computational techniques have proven to be an invaluable asset in unraveling the mysteries encoded within genomic sequences. As this research frontier continues to expand and evolve, we anticipate that the insights provided by this review will spur continued innovation and exploration, propelling us towards new discoveries in the dynamic world of genome data analysis.

## Figures and Tables

**Table 1:** Overview of Applications of Transformer Architecture and Attention Mechanism for Genome Data.

Research Topic	Studies
**Sequence and Site Prediction**	Raad et al. [34], Shen et al. [35], Li et al. [36], Ma et al. [37], Zeng et al. [38], Mai et al. [39], Song et al. [40], Tang et al. [41], Du et al. [42], Pan et al. [43], Zhuang et al. [44], Huang et al. [45], Guan et al. [46], Li et al. [47], Liu et al. [48], Du et al. [49], Wenjing et al. [50], Cao et al. [51], He et al. [52], Shen et al. [53], Zhang et al. [54], Jiang et al. [55], Bhukya et al. [56], Muneer et al. [57], Wekesa et al. [58], Liang et al. [59], Zhang et al. [60], Xie et al. [61], Fan et al. [62], Tsukiyama et al. [63], Gao et al. [64], Ullah et al. [65], Guo et al. [66], Wang et al. [67], Sun et al. [68]
**Gene Expression and Phenotype Prediction**	Zhang et al. [69], Lee et al. [70], Chen et al. [71], Kang et al. [72], Liao et al. [73], Angenent-Mari et al. [74], Zuo et al. [75], Karbalayghareh et al. [76], Pham et al. [77], Dominic et al. [78], Lee et al. [79], Li et al. [80], Bu et al. [81], Schapke et al. [82]
**ncRNA and circRNA Studies**	Liu et al. [83], Song et al. [84], Wekesa et al. [85], Wu et al. [86], Yang et al. [87], Liu et al. [88], Gao and Shang [89], Yuan and Yang [90], Song et al. [91], Chen et al. [92], Yang et al. [93], Li et al. [94], Wang et al. [95], Li et al. [96], Fan et al. [97], Sheng et al. [98], Niu et al. [99], Zhang et al. [100], Liu et al. [101], Guo et al. [102]
**Transcription Process Insights**	Clauwaert et al. [103], Feng et al. [104], Han et al. [105], Tao et al. [106], Asim et al. [107], Park et al. [108], Yan et al. [109], Song et al. [110]
**Multi-omics/modal Tasks**	Gong et al. [111], Kayikci and Khoshgoftaar [112], Ye et al. [113], Kang et al. [114], Wang et al. [115], Chan et al. [116]
**CRISPR Efficacy and Outcome Prediction**	Liu et al. [117], Liu et al. [118], Wan and Jiang [119], Xiao et al. [120], Mathis et al. [121], Zhang et al. [122], Zhang et al. [123]
**Gene Regulatory Network Inference**	Lin and Ou-Yang [124], Xu et al. [125], Feng et al. [126], Ullah and Ben-Hur [127], Xie et al. [128]
**Disease Prognosis Estimation**	Lee [129], Choi and Lee [130], Dutta et al. [131], Xing et al. [132], Meng et al. [133], Feng et al. [134]
**Gene Expression-based Classification**	Gokhale et al. [135], Beykikhoshk et al. [136], Manica et al. [137], Lee et al. [138]
**Proteomics**	Hou et al. [139], Gong et al. [140], Armenteros et al. [141], Littmann et al. [142]
**Cell-Type Identification**	Song et al. [143], Feng et al. [144], Buterez et al. [145], Zhang et al. [146]
**Predicting Drug-Drug Interactions**	Schwarz et al. [147], Kim et al. [148], Liu and Xie [149], Wang et al. [150]
**Other Topics**	Yu et al. [151], Yamaguchi and Saito [152], Zhou et al. [153], Cao et al. [154], Gupta and Shankar [155], Zhang et al. [156], Choi and Chae [157]

**Table 2:** Distribution of Published Articles across Different Journals.

Journal	Counts	Percentage (%)
Briefings in Bioinformatics	20	16.1
Bioinformatics	9	7.3
BMC Bioinformatics	9	7.3
Frontiers in Genetics	9	7.3
IEEE-ACM Transactions on Computational Biology and Bioinformatics	7	5.6
PLOS Computational Biology	4	3.2
Nature Communications	4	3.2
Interdisciplinary Sciences-Computational Life Sciences	4	3.2
Computational and Structural Biotechnology Journal	3	2.4
Scientific Reports	3	2.4
Biology-Basel	2	1.6
Mathematical Biosciences and Engineering	2	1.6
Frontiers in Oncology	2	1.6
Computational Biology and Chemistry	2	1.6
Proceedings of the National Academy of Sciences of the United States of America	2	1.6
Methods	2	1.6
Nucleic Acids Research	2	1.6
Cells	2	1.6
Frontiers in Cell and Developmental Biology	2	1.6
Others (<2 Publications)	34	27.4

## Data Availability

No new data were created or analyzed in this study.

## References

[1] Auslander N., Gussow A.B., Koonin E.V. (2021). Incorporating Machine Learning into Established Bioinformatics Frameworks. Int. J. Mol. Sci..

[2] Lee M. (2023). Deep Learning Techniques with Genomic Data in Cancer Prognosis: A Comprehensive Review of the 2021–2023 Literature. Biology.

[3] Gomes R., Paul N., He N., Huber A.F., Jansen R.J. (2022). Application of Feature Selection and Deep Learning for Cancer Prediction Using DNA Methylation Markers. Genes.

[4] Sadad T., Aurangzeb R.A., Safran M., Imran, Alfarhood S., Kim J. (2023). Classification of Highly Divergent Viruses from DNA/RNA Sequence Using Transformer-Based Models. Biomedicines.

[5] Lee M. (2023). Recent Advances in Deep Learning for Protein-Protein Interaction Analysis: A Comprehensive Review. Molecules.

[6] Kim Y., Lee M. (2023). Deep Learning Approaches for lncRNA-Mediated Mechanisms: A Comprehensive Review of Recent Developments. Int. J. Mol. Sci..

[7] Brown T., Mann B., Ryder N., Subbiah M., Kaplan J.D., Dhariwal P., Neelakantan A., Shyam P., Sastry G., Askell A. (2020). Language models are few-shot learners. Adv. Neural Inf. Process. Syst..

[8] Khan S., Naseer M., Hayat M., Zamir S.W., Khan F.S., Shah M. (2022). Transformers in vision: A survey. ACM Comput. Surv. (CSUR).

[9] Liu Z., Lin Y., Cao Y., Hu H., Wei Y., Zhang Z., Lin S., Guo B. Swin transformer: Hierarchical vision transformer using shifted windows. Proceedings of the IEEE/CVF International Conference on Computer Vision.

[10] Han K., Wang Y., Chen H., Chen X., Guo J., Liu Z., Tang Y., Xiao A., Xu C., Xu Y. (2022). A survey on vision transformer. IEEE Trans. Pattern Anal. Mach. Intell..

[11] Vaswani A., Shazeer N., Parmar N., Uszkoreit J., Jones L., Gomez A.N., Kaiser Ł., Polosukhin I. (2017). Attention is all you need. Adv. Neural Inf. Process. Syst..

[12] Wei Z., Yan Q., Lu X., Zheng Y., Sun S., Lin J. (2023). Compression Reconstruction Network with Coordinated Self-Attention and Adaptive Gaussian Filtering Module. Mathematics.

[13] Jin A., Zeng X. (2023). A Novel Deep Learning Method for Underwater Target Recognition Based on Res-Dense Convolutional Neural Network with Attention Mechanism. J. Mar. Sci. Eng..

[14] Gao L., Wu Y., Yang T., Zhang X., Zeng Z., Chan C.K.D., Chen W. (2023). Research on Image Classification and Retrieval Using Deep Learning with Attention Mechanism on Diaspora Chinese Architectural Heritage in Jiangmen, China. Buildings.

[15] Lu J., Ren H., Shi M., Cui C., Zhang S., Emam M., Li L. (2023). A Novel Hybridoma Cell Segmentation Method Based on Multi-Scale Feature Fusion and Dual Attention Network. Electronics.

[16] Cheng S., Liu Y. (2023). Research on Transportation Mode Recognition Based on Multi-Head Attention Temporal Convolutional Network. Sensors.

[17] Kasgari A.B., Safavi S., Nouri M., Hou J., Sarshar N.T., Ranjbarzadeh R. (2023). Point-of-Interest Preference Model Using an Attention Mechanism in a Convolutional Neural Network. Bioengineering.

[18] Raimundo A., Pavia J.P., Sebastião P., Postolache O. (2023). YOLOX-Ray: An Efficient Attention-Based Single-Staged Object Detector Tailored for Industrial Inspections. Sensors.

[19] Kim T., Pak W. (2023). Deep Learning-Based Network Intrusion Detection Using Multiple Image Transformers. Appl. Sci..

[20] Feng S., Zhu X., Ma S., Lan Q. (2023). GIT: A Transformer-Based Deep Learning Model for Geoacoustic Inversion. J. Mar. Sci. Eng..

[21] Jiang D., Shi G., Li N., Ma L., Li W., Shi J. (2023). TRFM-LS: Transformer-Based Deep Learning Method for Vessel Trajectory Prediction. J. Mar. Sci. Eng..

[22] Cao L., Wang Q., Hong J., Han Y., Zhang W., Zhong X., Che Y., Ma Y., Du K., Wu D. (2023). MVI-TR: A Transformer-Based Deep Learning Model with Contrast-Enhanced CT for Preoperative Prediction of Microvascular Invasion in Hepatocellular Carcinoma. Cancers.

[23] Shrestha A., Mahmood A. (2019). Review of deep learning algorithms and architectures. IEEE Access.

[24] Bhatt D., Patel C., Talsania H., Patel J., Vaghela R., Pandya S., Modi K., Ghayvat H. (2021). CNN variants for computer vision: History, architecture, application, challenges and future scope. Electronics.

[25] Yu Y., Si X., Hu C., Zhang J. (2019). A review of recurrent neural networks: LSTM cells and network architectures. Neural Comput..

[26] Lee M., Seok J. (2019). Controllable generative adversarial network. IEEE Access.

[27] Kim J., Lee M. (2023). Portfolio optimization using predictive auxiliary classifier generative adversarial networks. Eng. Appl. Artif. Intell..

[28] Lee M., Seok J. (2022). Score-guided generative adversarial networks. Axioms.

[29] Lee M., Seok J. (2021). Estimation with uncertainty via conditional generative adversarial networks. Sensors.

[30] Yeom T., Lee M. (2023). DuDGAN: Improving Class-Conditional GANs via Dual-Diffusion. arXiv.

[31] Ko K., Lee M. (2023). ZIGNeRF: Zero-shot 3D Scene Representation with Invertible Generative Neural Radiance Fields. arXiv.

[32] Lee M. (2023). Recent Advances in Generative Adversarial Networks for Gene Expression Data: A Comprehensive Review. Mathematics.

[33] Niu Z., Zhong G., Yu H. (2021). A review on the attention mechanism of deep learning. Neurocomputing.

[34] Raad J., Bugnon L.A., Milone D.H., Stegmayer G. (2022). miRe2e: A full end-to-end deep model based on transformers for prediction of pre-miRNAs. Bioinformatics.

[35] Shen Z., Zhang Q., Han K., Huang D.S. (2022). A Deep Learning Model for RNA-Protein Binding Preference Prediction Based on Hierarchical LSTM and Attention Network. IEEE-ACM Trans. Comput. Biol. Bioinform..

[36] Li Q., Cheng X., Song C., Liu T. (2023). M6A-BERT-Stacking: A Tissue-Specific Predictor for Identifying RNA N6-Methyladenosine Sites Based on BERT and Stacking Strategy. Symmetry.

[37] Ma Z.W., Zhao J.P., Tian J., Zheng C.H. (2022). DeeProPre: A promoter predictor based on deep learning. Comput. Biol. Chem..

[38] Zeng R., Cheng S., Liao M. (2021). 4mCPred-MTL: Accurate Identification of DNA 4mC Sites in Multiple Species Using Multi-Task Deep Learning Based on Multi-Head Attention Mechanism. Front. Cell Dev. Biol..

[39] Mai D.H.A., Nguyen L.T., Lee E.Y. (2022). TSSNote-CyaPromBERT: Development of an integrated platform for highly accurate promoter prediction and visualization of Synechococcus sp. and Synechocystis sp. through a state-of-the-art natural language processing model BERT. Front. Genet..

[40] Song J., Tian S., Yu L., Yang Q., Xing Y., Zhang C., Dai Q., Duan X. (2022). MD-MLI: Prediction of miRNA-lncRNA Interaction by Using Multiple Features and Hierarchical Deep Learning. IEEE-ACM Trans. Comput. Biol. Bioinform..

[41] Tang X., Zheng P., Li X., Wu H., Wei D.Q., Liu Y., Huang G. (2022). Deep6mAPred: A CNN and Bi-LSTM-based deep learning method for predicting DNA N6-methyladenosine sites across plant species. Methods.

[42] Du B., Liu Z., Luo F. (2022). Deep multi-scale attention network for RNA-binding proteins prediction. Inf. Sci..

[43] Pan Z., Zhou S., Zou H., Liu C., Zang M., Liu T., Wang Q. (2023). CRMSNet: A deep learning model that uses convolution and residual multi-head self-attention block to predict RBPs for RNA sequence. Proteins-Struct. Funct. Bioinform..

[44] Zhuang J., Liu D., Lin M., Qiu W., Liu J., Chen S. (2021). PseUdeep: RNA Pseudouridine Site Identification with Deep Learning Algorithm. Front. Genet..

[45] Huang Y., Luo J., Jing R., Li M. (2022). Multi-model predictive analysis of RNA solvent accessibility based on modified residual attention mechanism. Brief. Bioinform..

[46] Guan X., Wang Y., Shao W., Li Z., Huang S., Zhang D. (2022). S2Snet: Deep learning for low molecular weight RNA identification with nanopore. Brief. Bioinform..

[47] Li X., Zhang S., Shi H. (2022). An improved residual network using deep fusion for identifying RNA 5-methylcytosine sites. Bioinformatics.

[48] Fei Y., Zhang H., Wang Y., Liu Z., Liu Y. (2022). LTPConstraint: A transfer learning based end-to-end method for RNA secondary structure prediction. BMC Bioinform..

[49] Du Z., Xiao X., Uversky V.N. (2022). DeepA-RBPBS: A hybrid convolution and recurrent neural network combined with attention mechanism for predicting RBP binding site. J. Biomol. Struct. Dyn..

[50] Wenjing Y., Baoyu Z., Min Z., Qingchuan Z., Hong W., Da M. (2022). AttentionSplice: An Interpretable Multi-Head Self-Attention Based Hybrid Deep Learning Model in Splice Site Prediction. Chin. J. Electron..

[51] Cao L., Liu P., Chen J., Deng L. (2022). Prediction of Transcription Factor Binding Sites Using a Combined Deep Learning Approach. Front. Oncol..

[52] He S., Gao B., Sabnis R., Sun Q. (2023). RNAdegformer: Accurate prediction of mRNA degradation at nucleotide resolution with deep learning. Brief. Bioinform..

[53] Shen L.C., Liu Y., Song J., Yu D.J. (2021). SAResNet: Self-attention residual network for predicting DNA-protein binding. Brief. Bioinform..

[54] Zhang Y., Wang Z., Zeng Y., Zhou J., Zou Q. (2021). High-resolution transcription factor binding sites prediction improved performance and interpretability by deep learning method. Brief. Bioinform..

[55] Jiang J.Y., Ju C.J.T., Hao J., Chen M., Wang W. (2021). JEDI: Circular RNA prediction based on junction encoders and deep interaction among splice sites. Bioinformatics.

[56] Bhukya R., Kumari A., Dasari C.M., Amilpur S. (2022). An attention-based hybrid deep neural networks for accurate identification of transcription factor binding sites. Neural Comput. Appl..

[57] Muneer A., Fati S.M., Akbar N.A., Agustriawan D., Wahyudi S.T. (2022). iVaccine-Deep: Prediction of COVID-19 mRNA vaccine degradation using deep learning. J. King Saud-Univ.-Comput. Inf. Sci..

[58] Wekesa J.S., Meng J., Luan Y. (2020). Multi-feature fusion for deep learning to predict plant lncRNA-protein interaction. Genomics.

[59] Liang J., Cui Z., Wu C., Yu Y., Tian R., Xie H., Jin Z., Fan W., Xie W., Huang Z. (2021). DeepEBV: A deep learning model to predict Epstein-Barr virus (EBV) integration sites. Bioinformatics.

[60] Zhang H., Fang J., Sun Y., Xie G., Lin Z., Gu G. (2023). Predicting miRNA-Disease Associations via Node-Level Attention Graph Auto-Encoder. IEEE-ACM Trans. Comput. Biol. Bioinform..

[61] Xie X., Wang Y., He K., Sheng N. (2023). Predicting miRNA-disease associations based on PPMI and attention network. BMC Bioinform..

[62] Fan X.Q., Hu J., Tang Y.X., Jia N.X., Yu D.J., Zhang G.J. (2022). Predicting RNA solvent accessibility from multi-scale context feature via multi-shot neural network. Anal. Biochem..

[63] Tsukiyama S., Hasan M.M., Deng H.W., Kurata H. (2022). BERT6mA: Prediction of DNA N6-methyladenine site using deep learning-based approaches. Brief. Bioinform..

[64] Gao Y., Chen Y., Feng H., Zhang Y., Yue Z. (2022). RicENN: Prediction of Rice Enhancers with Neural Network Based on DNA Sequences. Interdiscip.-Sci.-Comput. Life Sci..

[65] Ullah A., Malik K.M., Saudagar A.K.J., Khan M.B., Abul Hasanat M.H., AlTameem A., AlKhathami M., Sajjad M. (2022). COVID-19 Genome Sequence Analysis for New Variant Prediction and Generation. Mathematics.

[66] Guo Y., Zhou D., Li W., Cao J., Nie R., Xiong L., Ruan X. (2021). Identifying polyadenylation signals with biological embedding via self-attentive gated convolutional highway networks. Appl. Soft Comput..

[67] Wang Y., Hou Z., Yang Y., Wong K.c., Li X. (2022). Genome-wide identification and characterization of DNA enhancers with a stacked multivariate fusion framework. PLoS Comput. Biol..

[68] Sun L., Xu K., Huang W., Yang Y.T., Li P., Tang L., Xiong T., Zhang Q.C. (2021). Predicting dynamic cellular protein-RNA interactions by deep learning using in vivo RNA structures. Cell Res..

[69] Zhang T.H., Hasib M.M., Chiu Y.C., Han Z.F., Jin Y.F., Flores M., Chen Y., Huang Y. (2022). Transformer for Gene Expression Modeling (T-GEM): An Interpretable Deep Learning Model for Gene Expression-Based Phenotype Predictions. Cancers.

[70] Lee D., Yang J., Kim S. (2022). Learning the histone codes with large genomic windows and three-dimensional chromatin interactions using transformer. Nat. Commun..

[71] Chen Y., Xie M., Wen J. (2022). Predicting gene expression from histone modifications with self-attention based neural networks and transfer learning. Front. Genet..

[72] Kang M., Lee S., Lee D., Kim S. (2020). Learning Cell-Type-Specific Gene Regulation Mechanisms by Multi-Attention Based Deep Learning With Regulatory Latent Space. Front. Genet..

[73] Liao Y., Guo H., Jing R., Luo J., Li M., Li Y. (2021). Predicting gene expression levels from histone modification profiles by a hybrid deep learning network. Chemom. Intell. Lab. Syst..

[74] Angenent-Mari N.M., Garruss A.S., Soenksen L.R., Church G., Collins J.J. (2020). A deep learning approach to programmable RNA switches. Nat. Commun..

[75] Zuo Z., Wang P., Chen X., Tian L., Ge H., Qian D. (2021). SWnet: A deep learning model for drug response prediction from cancer genomic signatures and compound chemical structures. BMC Bioinform..

[76] Karbalayghareh A., Sahin M., Leslie C.S. (2022). Chromatin interaction-aware gene regulatory modeling with graph attention networks. Genome Res..

[77] Pham T.H., Qiu Y., Zeng J., Xie L., Zhang P. (2021). A deep learning framework for high-throughput mechanism-driven phenotype compound screening and its application to COVID-19 drug repurposing. Nat. Mach. Intell..

[78] Dominic N., Cenggoro T.W., Budiarto A., Pardamean B. (2022). Deep polygenic neural network for predicting and identifying yield-associated genes in Indonesian rice accessions. Sci. Rep..

[79] Lee H., Yeom S., Kim S. (2020). BP-GAN: Interpretable Human Branchpoint Prediction Using Attentive Generative Adversarial Networks. IEEE Access.

[80] Li Z., Zhong T., Huang D., You Z.H., Nie R. (2022). Hierarchical graph attention network for miRNA-disease association prediction. Mol. Ther..

[81] Bu Y., Jia C., Guo X., Li F., Song J. (2023). COPPER: An ensemble deep-learning approach for identifying exclusive virus-derived small interfering RNAs in plants. Briefings Funct. Genom..

[82] Schapke J., Tavares A., Recamonde-Mendoza M. (2022). EPGAT: Gene Essentiality Prediction With Graph Attention Networks. IEEE-ACM Trans. Comput. Biol. Bioinform..

[83] Liu Y., Yu Y., Zhao S. (2022). Dual Attention Mechanisms and Feature Fusion Networks Based Method for Predicting LncRNA-Disease Associations. Interdiscip.-Sci.-Comput. Life Sci..

[84] Song J., Tian S., Yu L., Yang Q., Dai Q., Wang Y., Wu W., Duan X. (2022). RLF-LPI: An ensemble learning framework using sequence information for predicting lncRNA-protein interaction based on AE-ResLSTM and fuzzy decision. Math. Biosci. Eng..

[85] Wekesa J.S., Meng J., Luan Y. (2020). A deep learning model for plant lncRNA-protein interaction prediction with graph attention. Mol. Genet. Genom..

[86] Wu H., Pan X., Yang Y., Shen H.B. (2021). Recognizing binding sites of poorly characterized RNA-binding proteins on circular RNAs using attention Siamese network. Brief. Bioinform..

[87] Yang T.H., Shiue S.C., Chen K.Y., Tseng Y.Y., Wu W.S. (2021). Identifying piRNA targets on mRNAs in C. elegans using a deep multi-head attention network. BMC Bioinform..

[88] Liu T., Zou B., He M., Hu Y., Dou Y., Cui T., Tan P., Li S., Rao S., Huang Y. (2022). LncReader: Identification of dual functional long noncoding RNAs using a multi-head self-attention mechanism. Brief. Bioinform..

[89] Gao M., Shang X. (2023). Identification of associations between lncRNA and drug resistance based on deep learning and attention mechanism. Front. Microbiol..

[90] Yuan L., Yang Y. (2021). DeCban: Prediction of circRNA-RBP Interaction Sites by Using Double Embeddings and Cross-Branch Attention Networks. Front. Genet..

[91] Song Z., Huang D., Song B., Chen K., Song Y., Liu G., Su J., de Magalhaes J.P., Rigden D.J., Meng J. (2021). Attention-based multi-label neural networks for integrated prediction and interpretation of twelve widely occurring RNA modifications. Nat. Commun..

[92] Chen K., Zhu X., Wang J., Hao L., Liu Z., Liu Y. (2023). ncDENSE: A novel computational method based on a deep learning framework for non-coding RNAs family prediction. BMC Bioinform..

[93] Yang Y., Hou Z., Ma Z., Li X., Wong K.C. (2021). iCircRBP-DHN: Identification of circRNA-RBP interaction sites using deep hierarchical network. Brief. Bioinform..

[94] Li G., Wang D., Zhang Y., Liang C., Xiao Q., Luo J. (2022). Using Graph Attention Network and Graph Convolutional Network to Explore Human CircRNA-Disease Associations Based on Multi-Source Data. Front. Genet..

[95] Wang H., Han J., Li H., Duan L., Liu Z., Cheng H. (2023). CDA-SKAG: Predicting circRNA-disease associations using similarity kernel fusion and an attention-enhancing graph autoencoder. Math. Biosci. Eng..

[96] Li G., Lin Y., Luo J., Xiao Q., Liang C. (2022). GGAECDA: Predicting circRNA-disease associations using graph autoencoder based on graph representation learning. Comput. Biol. Chem..

[97] Fan Y., Chen M., Pan X. (2022). GCRFLDA: Scoring lncRNA-disease associations using graph convolution matrix completion with conditional random field. Brief. Bioinform..

[98] Sheng N., Cui H., Zhang T., Xuan P. (2021). Attentional multi-level representation encoding based on convolutional and variance autoencoders for lncRNA-disease association prediction. Brief. Bioinform..

[99] Niu M., Zou Q., Lin C. (2022). CRBPDL: Identification of circRNA-RBP interaction sites using an ensemble neural network approach. PLoS Comput. Biol..

[100] Zhang Y., Ye F., Gao X. (2022). MCA-Net: Multi-Feature Coding and Attention Convolutional Neural Network for Predicting lncRNA-Disease Association. IEEE-ACM Trans. Comput. Biol. Bioinform..

[101] Liu Y., Fu Q., Peng X., Zhu C., Liu G., Liu L. (2021). Attention-Based Deep Multiple-Instance Learning for Classifying Circular RNA and Other Long Non-Coding RNA. Genes.

[102] Guo Y., Lei X., Liu L., Pan Y. (2023). circ2CBA: Prediction of circRNA-RBP binding sites combining deep learning and attention mechanism. Front. Comput. Sci..

[103] Clauwaert J., Menschaert G., Waegeman W. (2021). Explainability in transformer models for functional genomics. Brief. Bioinform..

[104] Feng P., Xiao A., Fang M., Wan F., Li S., Lang P., Zhao D., Zeng J. (2021). A machine learning-based framework for modeling transcription elongation. Proc. Natl. Acad. Sci. USA.

[105] Han K., Shen L.C., Zhu Y.H., Xu J., Song J., Yu D.J. (2022). MAResNet: Predicting transcription factor binding sites by combining multi-scale bottom-up and top-down attention and residual network. Brief. Bioinform..

[106] Tao Y., Ma X., Palmer D., Schwartz R., Lu X., Osmanbeyoglu H.U. (2022). Interpretable deep learning for chromatin-informed inference of transcriptional programs driven by somatic alterations across cancers. Nucleic Acids Res..

[107] Asim M.N., Ibrahim M.A., Malik M.I., Zehe C., Cloarec O., Trygg J., Dengel A., Ahmed S. (2022). EL-RMLocNet: An explainable LSTM network for RNA-associated multi-compartment localization prediction. Comput. Struct. Biotechnol. J..

[108] Park S., Koh Y., Jeon H., Kim H., Yeo Y., Kang J. (2020). Enhancing the interpretability of transcription factor binding site prediction using attention mechanism. Sci. Rep..

[109] Yan Z., Lecuyer E., Blanchette M. (2019). Prediction of mRNA subcellular localization using deep recurrent neural networks. Bioinformatics.

[110] Song J., Tian S., Yu L., Xing Y., Yang Q., Duan X., Dai Q. (2020). AC-Caps: Attention Based Capsule Network for Predicting RBP Binding Sites of LncRNA. Interdiscip.-Sci.-Comput. Life Sci..

[111] Gong P., Cheng L., Zhang Z., Meng A., Li E., Chen J., Zhang L. (2023). Multi-omics integration method based on attention deep learning network for biomedical data classification. Comput. Methods Prog. Biomed..

[112] Kayikci S., Khoshgoftaar T.M. (2023). Breast cancer prediction using gated attentive multimodal deep learning. J. Big Data.

[113] Ye L., Zhang Y., Yang X., Shen F., Xu B. (2021). An Ovarian Cancer Susceptible Gene Prediction Method Based on Deep Learning Methods. Front. Cell Dev. Biol..

[114] Kang Q., Meng J., Shi W., Luan Y. (2021). Ensemble Deep Learning Based on Multi-level Information Enhancement and Greedy Fuzzy Decision for Plant miRNA-lncRNA Interaction Prediction. Interdiscip.-Sci.-Comput. Life Sci..

[115] Wang C., Lye X., Kaalia R., Kumar P., Rajapakse J.C. (2022). Deep learning and multi-omics approach to predict drug responses in cancer. BMC Bioinform..

[116] Chan Y.H., Wang C., Soh W.K., Rajapakse J.C. (2022). Combining Neuroimaging and Omics Datasets for Disease Classification Using Graph Neural Networks. Front. Neurosci..

[117] Liu Q., He D., Xie L. (2019). Prediction of off-target specificity and cell-specific fitness of CRISPR-Cas System using attention boosted deep learning and network-based gene feature. PLoS Comput. Biol..

[118] Liu X., Wang S., Ai D. (2022). Predicting CRISPR/Cas9 Repair Outcomes by Attention-Based Deep Learning Framework. Cells.

[119] Wan Y., Jiang Z. (2023). TransCrispr: Transformer Based Hybrid Model for Predicting CRISPR/Cas9 Single Guide RNA Cleavage Efficiency. IEEE-ACM Trans. Comput. Biol. Bioinform..

[120] Xiao L.M., Wan Y.Q., Jiang Z.R. (2021). AttCRISPR: A spacetime interpretable model for prediction of sgRNA on-target activity. BMC Bioinform..

[121] Mathis N., Allam A., Kissling L., Marquart K.F., Schmidheini L., Solari C., Balazs Z., Krauthammer M., Schwank G. (2023). Predicting prime editing efficiency and product purity by deep learning. Nat. Biotechnol..

[122] Zhang Z.R., Jiang Z.R. (2022). Effective use of sequence information to predict CRISPR-Cas9 off-target. Comput. Struct. Biotechnol. J..

[123] Zhang G., Zeng T., Dai Z., Dai X. (2021). Prediction of CRISPR/Cas9 single guide RNA cleavage efficiency and specificity by attention-based convolutional neural networks. Comput. Struct. Biotechnol. J..

[124] Lin Z., Ou-Yang L. (2022). Inferring gene regulatory networks from single-cell gene expression data via deep multi-view contrastive learning. Brief. Bioinform..

[125] Xu J., Zhang A., Liu F., Zhang X. (2023). STGRNS: An interpretable transformer-based method for inferring gene regulatory networks from single-cell transcriptomic data. Bioinformatics.

[126] Feng X., Fang F., Long H., Zeng R., Yao Y. (2022). Single-cell RNA-seq data analysis using graph autoencoders and graph attention networks. Front. Genet..

[127] Ullah F., Ben-Hur A. (2021). A self-attention model for inferring cooperativity between regulatory features. Nucleic Acids Res..

[128] Xie X., Wang Y., Sheng N., Zhang S., Cao Y., Fu Y. (2022). Predicting miRNA-disease associations based on multi-view information fusion. Front. Genet..

[129] Lee M. (2022). An Ensemble Deep Learning Model with a Gene Attention Mechanism for Estimating the Prognosis of Low-Grade Glioma. Biology.

[130] Choi S.R., Lee M. (2022). Estimating the Prognosis of Low-Grade Glioma with Gene Attention Using Multi-Omics and Multi-Modal Schemes. Biology.

[131] Dutta P., Patra A.P., Saha S. (2022). DeePROG: Deep Attention-Based Model for Diseased Gene Prognosis by Fusing Multi-Omics Data. IEEE-ACM Trans. Comput. Biol. Bioinform..

[132] Xing X., Yang F., Li H., Zhang J., Zhao Y., Gao M., Huang J., Yao J. (2022). Multi-level attention graph neural network based on co-expression gene modules for disease diagnosis and prognosis. Bioinformatics.

[133] Meng X., Wang X., Zhang X., Zhang C., Zhang Z., Zhang K., Wang S. (2022). A Novel Attention-Mechanism Based Cox Survival Model by Exploiting Pan-Cancer Empirical Genomic Information. Cells.

[134] Feng C., Xiang T., Yi Z., Meng X., Chu X., Huang G., Zhao X., Chen F., Xiong B., Feng J. (2021). A Deep-Learning Model With the Attention Mechanism Could Rigorously Predict Survivals in Neuroblastoma. Front. Oncol..

[135] Gokhale M., Mohanty S.K., Ojha A. (2023). GeneViT: Gene Vision Transformer with Improved DeepInsight for cancer classification. Comput. Biol. Med..

[136] Beykikhoshk A., Quinn T.P., Lee S.C., Tran T., Venkatesh S. (2020). DeepTRIAGE: Interpretable and individualised biomarker scores using attention mechanism for the classification of breast cancer sub-types. BMC Med. Genom..

[137] Manica M., Oskooei A., Born J., Subramanian V., Saez-Rodriguez J., Martinez M.R. (2019). Toward Explainable Anticancer Compound Sensitivity Prediction via Multimodal Attention-Based Convolutional Encoders. Mol. Pharm..

[138] Lee S., Lim S., Lee T., Sung I., Kim S. (2020). Cancer subtype classification and modeling by pathway attention and propagation. Bioinformatics.

[139] Hou Z., Yang Y., Li H., Wong K.C., Li X. (2021). iDeepSubMito: Identification of protein submitochondrial localization with deep learning. Brief. Bioinform..

[140] Gong H., Wen J., Luo R., Feng Y., Guo J., Fu H., Zhou X. (2023). Integrated mRNA sequence optimization using deep learning. Brief. Bioinform..

[141] Armenteros J.J.A., Salvatore M., Emanuelsson O., Winther O., von Heijne G., Elofsson A., Nielsen H. (2019). Detecting sequence signals in targeting peptides using deep learning. Life Sci. Alliance.

[142] Littmann M., Heinzinger M., Dallago C., Weissenow K., Rost B. (2021). Protein embeddings and deep learning predict binding residues for various ligand classes. Sci. Rep..

[143] Song T., Dai H., Wang S., Wang G., Zhang X., Zhang Y., Jiao L. (2022). TransCluster: A Cell-Type Identification Method for single-cell RNA-Seq data using deep learning based on transformer. Front. Genet..

[144] Feng X., Zhang H., Lin H., Long H. (2023). Single-cell RNA-seq data analysis based on directed graph neural network. Methods.

[145] Buterez D., Bica I., Tariq I., Andres-Terre H., Lio P. (2022). CellVGAE: An unsupervised scRNA-seq analysis workflow with graph attention networks. Bioinformatics.

[146] Zhang Y., Blanchette M. (2022). Reference panel guided topological structure annotation of Hi-C data. Nat. Commun..

[147] Schwarz K., Allam A., Gonzalez N.A.P., Krauthammer M. (2021). AttentionDDI: Siamese attention-based deep learning method for drug-drug interaction predictions. BMC Bioinform..

[148] Kim E., Nam H. (2022). DeSIDE-DDI: Interpretable prediction of drug-drug interactions using drug-induced gene expressions. J. Cheminform..

[149] Liu Q., Xie L. (2021). TranSynergy: Mechanism-driven interpretable deep neural network for the synergistic prediction and pathway deconvolution of drug combinations. PLoS Comput. Biol..

[150] Wang J., Liu X., Shen S., Deng L., Liu H. (2022). DeepDDS: Deep graph neural network with attention mechanism to predict synergistic drug combinations. Brief. Bioinform..

[151] Yu G., Zeng J., Wang J., Zhang H., Zhang X., Guo M. (2021). Imbalance deep multi-instance learning for predicting isoform-isoform interactions. Int. J. Intell. Syst..

[152] Yamaguchi H., Saito Y. (2021). Evotuning protocols for Transformer-based variant effect prediction on multi-domain proteins. Brief. Bioinform..

[153] Zhou J., Chen Q., Braun P.R., Mandell K.A.P., Jaffe A.E., Tan H.Y., Hyde T.M., Kleinman J.E., Potash J.B., Shinozaki G. (2022). Deep learning predicts DNA methylation regulatory variants in the human brain and elucidates the genetics of psychiatric disorders. Proc. Natl. Acad. Sci. USA.

[154] Cao L., Zhang Q., Song H., Lin K., Pang E. (2022). DeepASmRNA: Reference-free prediction of alternative splicing events with a scalable and interpretable deep learning model. iScience.

[155] Gupta S., Shankar R. (2023). miWords: Transformer-based composite deep learning for highly accurate discovery of pre-miRNA regions across plant genomes. Brief. Bioinform..

[156] Zhang Z.Y., Ning L., Ye X., Yang Y.H., Futamura Y., Sakurai T., Lin H. (2022). iLoc-miRNA: Extracellular/intracellular miRNA prediction using deep BiLSTM with attention mechanism. Brief. Bioinform..

[157] Choi J.M., Chae H. (2023). moBRCA-net: A breast cancer subtype classification framework based on multi-omics attention neural networks. BMC Bioinform..

[158] Yin C., Chen Z. (2020). Developing Sustainable Classification of Diseases via Deep Learning and Semi-Supervised Learning. Healthcare.

[159] Song H., Yin C., Li Z., Feng K., Cao Y., Gu Y., Sun H. (2023). Identification of Cancer Driver Genes by Integrating Multiomics Data with Graph Neural Networks. Metabolites.

[160] Song J.T., Woo D.U., Lee Y., Choi S.H., Kang Y.J. (2021). The Semi-Supervised Strategy of Machine Learning on the Gene Family Diversity to Unravel Resveratrol Synthesis. Plants.

[161] Munoz S.A., Park J., Stewart C.M., Martin A.M., Hedengren J.D. (2023). Deep Transfer Learning for Approximate Model Predictive Control. Processes.

[162] Dastour H., Hassan Q.K. (2023). A Comparison of Deep Transfer Learning Methods for Land Use and Land Cover Classification. Sustainability.

[163] Yang L., Huang R., Zhang J., Huang J., Wang L., Dong J., Shao J. (2023). Inter-Continental Transfer of Pre-Trained Deep Learning Rice Mapping Model and Its Generalization Ability. Remote Sens..

